# Deciphering the Significance of Platelet‐Derived Chloride Ion Channel Gene (BEST3) Through Platelet‐Related Subtypes Mining for Non‐Small Cell Lung Cancer

**DOI:** 10.1111/jcmm.70233

**Published:** 2024-12-21

**Authors:** Hanxiao Ren, Meng‐Ze Du, Yulin Liao, Ruiling Zu, Lubei Rao, Run Xiang, Xingmei Zhang, Shan Liu, Peiyin Zhang, Ping Leng, Ling Qi, Huaichao Luo

**Affiliations:** ^1^ College of Medical Technology Chengdu University of Traditional Chinese Medicine Chengdu Sichuan Province People's Republic of China; ^2^ Department of Clinical Laboratory, Sichuan Clinical Research Center for Cancer, Sichuan Cancer Hospital & Institute, Sichuan Cancer Center Affiliated Cancer Hospital of the University of Electronic Science and Technology of China Chengdu China; ^3^ School of Health and Medical Technology Chengdu Neusoft University Chengdu Sichuan Province People's Republic of China; ^4^ Department of Thoracic Surgery, Sichuan Cancer Hospital, Affiliate to the School of Medicine The University of Electronic Science and Technology of China Chengdu China; ^5^ Department of Core Medical Laboratory The Sixth Affiliated Hospital of Guangzhou Medical University, Qingyuan People's Hospital Qingyuan China

**Keywords:** BEST3, metastasis, NSCLC, platelets, prognosis, proliferation

## Abstract

This study investigates platelet‐related subtypes in non‐small cell lung cancer (NSCLC) and seeks to identify genes associated with prognosis, focusing on the clinical significance of the chloride ion channel gene BEST3. We utilised sequencing and clinical data from GEO, TCGA and the Xena platform, building a risk model based on genetic features. TCGA and GSE37745 served as training cohorts, while GSE50081, GSE13213, GSE30129 and GSE42127 were validation cohorts. Immunotherapy datasets (GSE135222, TCGA‐SKCM) were also analysed. Differentially expressed genes (DEGs) were identified using Limma, subtypes through ConsensusClusterPlus and key prognostic genes using COX regression, Random Forest and LASSO‐COX. BEST3 expression was validated by flow cytometry (FCM) and functional assays in A549 cells with lentiviral overexpression evaluated its impact on apoptosis, proliferation and migration. Three platelet‐related subtypes were identified, with ten key prognostic genes (including BEST3). Gene Ontology (GO) analysis showed six genes involved in platelet pathways. BEST3 was highly expressed in the platelet subtype 1. Flow cytometry confirmed elevated BEST3 levels in NSCLC (35.9% vs. 27.3% in healthy individuals). Overexpression of BEST3 in NSCLC cells suppressed apoptosis and promoted proliferation and migration. The discovery of three platelet subtypes and the role of BEST3 in promoting tumour growth and migration highlights its potential as a therapeutic target and prognostic marker in NSCLC.

**Abbreviations**
BEST3: Bestrophin‐3
CNV: copy number variation
DEGs: differentially expressed genes
LUAD: lung adenocarcinoma
LUSC: lung squamous cell carcinoma
NSCLC: non‐small cell lung cancer
OS: overall survival
PltDB: platelet expression atlas database
PRDEGs: platelet‐related differential expressed genes
PRGs: platelet‐related genes
PRSS: platelet‐related survival score
ssGSEA: single‐sample gene set enrichment analysis

## 1 Introduction

Lung cancer is one of the most commonly diagnosed cancers worldwide and is also the leading cause of cancer‐related deaths globally [1]. Among the subtypes of lung cancer, non‐small cell lung cancer (NSCLC) is the most common subtype, accounting for approximately 85% of all lung cancer cases [2]. About 75% of patients are diagnosed with lung cancer at an advanced stage, leading to a low 5‐year survival rate. NSCLC encompasses several cancer types, including lung adenocarcinoma (LUAD), lung squamous cell carcinoma (LUSC) and large cell carcinoma. Among these, LUAD and LUSC are the primary histological subtypes of NSCLC [3].

Lung cancer is a heterogeneous disease characterised by a wide range of clinical and pathological features. An increasing body of research has delved deep into the molecular and genomic levels of analysis for NSCLC to gain a better understanding of its heterogeneity. For instance, Chen et al. [4] have identified nine major genomic subtypes of NSCLC in the TCGA dataset at the molecular level. These subtypes consist of three genomic subtypes related to LUSC and six subtypes associated with LUAD. Li et al. [5] employed an innovative array‐based approach that harnessed the interactions between graphene oxide and gold nanoparticles modified with single‐stranded DNA (ssDNA‐AuNPs). By integrating features from the overall cellular proteome, they achieved subtype differentiation within lung cancer. Wilkerson et al. [6] and Hayes et al. [7] performed subtyping of LUAD and LUSC using mRNA microarrays and DNA microarrays. They successfully identified reproducible expression subtypes. The analyses conducted in these studies for NSCLC subtypes allow for improved patient stratification. This, in turn, leads to more precise prognostic insights, ultimately elevating the overall survival (OS) rates of cancer patients and offering more effective treatment strategies. As conventional treatments such as chemotherapy, radiotherapy, targeted therapy and immunotherapy gradually face the challenge of drug resistance, the search for new therapeutic strategies for NSCLC becomes increasingly urgent. Zou et al. [8]. focused on the mechanism of ferroptosis in NSCLC and explored how it is related to NSCLC through various signalling cascades. Their study highlights the potential application of ferroptosis in nanoparticle‐based immunotherapy, offering a novel therapeutic approach that could be applied in clinical treatment of NSCLC. Additionally, Hu et al. [9] investigated the potential mechanisms of ERβ‐induced cisplatin resistance, proposing an alternative treatment strategy that could enhance the efficacy of platinum‐based chemotherapy in NSCLC patients.

It is widely known that platelets play a crucial role in the pathophysiology of haemostasis, coagulation and thrombotic disorders. Beyond haemostasis, platelets are also involved in immune responses, cancer metastasis, angiogenesis, tissue inflammation and regeneration [10]. In addition, a growing body of research suggests direct or indirect interactions between platelets and tumour cells. These interactions contribute to the promotion of tumour cell growth and metastasis of malignant tumours and increase tumour cell plasticity [11]. These interactions facilitate stable adhesion between platelets and tumour cells, influencing tumour metastasis. Tumour cells employ various mechanisms to promote platelet activation, leading to platelet aggregation, thrombus formation and enhanced blood coagulation, which in turn strengthens adhesion to tumour cells [12, 13]. Given the critical role of platelets in cancer biology, we hypothesize that genes associated with platelets may play a significant role in the precise subtyping of NSCLC patients. With the rapid development of high‐throughput sequencing technology [14], it has become a highly sensitive approach in molecular biology to study and evaluate tumour development and prognosis in combination with multiple dataset analysis and database mining. Therefore, we hypothesize that genes associated with platelets may play a significant role in the precise subtyping of NSCLC patients.

The primary aim of this study is to employ bioinformatics tools to identify distinct platelet subtypes in NSCLC and to elucidate their associations with tumour characteristics and patient outcomes. By analysing differentially expressed genes (DEGs) among these subtypes, we aim to identify key prognostic genes. Additionally, we validated the abnormal expression of the BEST3 chloride ion channel gene in NSCLC through flow cytometry (FCM) and provided gene ontology enrichment analysis annotations. Overexpression of BEST3 significantly correlates with tumorigenesis, metastasis and poor prognosis in NSCLC patients. These findings underscore the potential of platelet‐related genes in refining NSCLC subtyping and prognostication, thereby offering new insights for therapeutic strategies and patient management.

## 2 Materials and Methods

### 2.1 Data Collection and Processing

Based on the GSE89843 dataset available in the public repository of the GEO database (https://www.ncbi.nlm.nih.gov/geo/), RNA sequencing data of platelets from patients with NSCLC can be obtained. This dataset comprises 402 platelet samples from NSCLC patients at various stages and 377 platelet samples from healthy individuals. This data will be used to identify DEGs in platelets between NSCLC patients and healthy individuals. Using the keyword ‘Platelet’ in the MSigDB database (https://www.gsea‐msigdb.org/gsea/msigdb), we retrieved a total of 37 GO pathways and REACTOME pathways related to platelets (Table S1). After taking the union of genes from all these pathways, we identified a total of 707 platelet‐related genes (PRGs). We downloaded a total of 988 samples, including normal tissue samples from the UCSC Xena website (UCSC Xena (xenabrowser.net)) and NSCLC samples from TCGA (https://www.cancer.gov/ccg/research/genome‐sequencing/tcga). These NSCLC samples were used to construct the training set and all of them come with survival time information. Additionally, we selected 181 tumour patient samples from the GSE50081 dataset from the GPL570 platform for validation of the prognostic model. These samples also contain survival time information.

### 2.2 Identification and Characterisation of Platelet‐Related Differentially Expressed Genes (PRDEGs)

In dataset GSE89843, we conducted an analysis of platelets from individuals with NSCLC and healthy controls using the Limma package. We applied specific criteria to identify DEGs, setting the selection criteria as FDR < 0.01 and |log_2_FC| > 1. Subsequently, we took the union of the DEGs with 707 genes related to platelets to create a set known as PRGs. The intersection of PRGs with differentially expressed genes within the tissue (TCGA) led to the identification of PRDEGs. Visualisation of the results involved creating volcano plots and Venn diagrams, which were generated using the ggplot2 package in R software (version 4.1.2; https://www.R‐project.org).

### 2.3 Construction of Platelet‐Related Survival Score Model (PRSS)

First, we performed subtype differentiation of NSCLC patients using the R package ConsensusClusterPlus. Subsequently, we utilised differential genes between subtypes and conducted a univariable Cox regression analysis, setting a threshold of 0.01 to filter prognostic genes. We employed the Random Forest algorithm from the randomForestSRC R package and the LASSO‐COX algorithm from the glmnet R package to individually screen for prognostic genes. Subsequently, we intersected the prognostic genes identified by both algorithms, yielding the set of prognostic feature genes for our model. Finally, the regression coefficients for the model's feature genes were computed using multivariable Cox regression analysis. Subsequently, patient scores were calculated based on the formula:

$$\text{Score}=\sum_{i=0}^{n}\mathrm{\beta i}*\mathrm{\chi i}$$

*βi*: weighting coefficient of each gene; *χi*: expression of each gene.

Based on the median score, the patients were categorised into high and low‐scoring groups. Survival analysis was performed using the R package survminer to assess the different prognoses of the high and low scoring groups. The prognostic efficacy of the training and validation sets was analysed by calculating the AUC of the time‐dependent ROC using the R package timeROC.

### 2.4 Immune Infiltration Analysis

The infiltration of individual immune cells is determined based on the single‐sample gene set enrichment analysis (ssGSEA) algorithm in the GSVA package in R software using default reference values. In the ssGSEA algorithm, a rank‐based approach calculates a score representing the absolute enrichment of a specific genome in the sample and the degree of inflammatory infiltration of different types of immune cells in samples from various patients [15]. We analysed data sets from different genomes and cell expression signature files from Pornpimol et al. [16].

### 2.5 Platelet Preparation

Peripheral venous blood was obtained from lung cancer patients in the inpatient department of Sichuan Cancer Hospital and healthy physical examination subjects. Platelet‐rich plasma (PRP) was obtained by centrifugation of citrated peripheral blood specimens at 120 × g for 20 min. The isolation method used in this study was Best, Myron et al. [17, 18]. Platelets are prepared at room temperature. Subsequently, the PRP was partitioned into the monopositive and dipositive groups. Monopositive group was added with APC‐coupled Bestrophin 3 Antibody (OTI2H3) (1:250); Dipositive group was added with two fluorescently labelled monoclonal antibodies (PE‐coupled anti‐CD62‐P (BD Pharmingen, CA) (1:250) and APC‐linked Bestrophin 3 Antibody (OTI2H3) (1:250)). Specific methods for the preparation of activated platelets ref. [19].

### 2.6 Flow Cytometric Analysis

Firstly, 2 μL of APC‐coupled Bestrophin 3 Antibody (OTI2H3) (1:250) was added to the group A antigen detection tube, followed by 5 μL of PRP, and 500 μL of phosphate‐buffered saline (PBS) buffer was added after incubation for 15–20 min at room temperature, followed by detection by FCM. Next, 2 μL each of two fluorescently labelled monoclonal antibodies (PE‐coupled anti‐CD62‐P (BD Pharmingen, CA) (1:250) and APC‐labelled Bestrophin 3 Antibody (OTI2H3) (1:250)) were injected into the antigen detection tube of group B. 5 μL of PRP was added and incubated at room temperature for 15–20 min. 500 μL of phosphate‐buffered saline (PBS) buffer was added and detected by FCM. All were unaffected by light. Isotype‐matched antibodies control non‐specific antibody binding. Platelets were distinguished by specific binding of anti‐Bestrophin 3 and characteristic forward and side scattering [20]. Analysis was performed using a BDFACSCantoll flow cytometer (BD Biosciences, CA).

### 2.7 Cell Culture

A549 cells were maintained at 37°C in a 5% CO₂ incubator (Thermo Fisher Scientific). Cells were routinely cultured in DMEM (Gibco, #C11995500BT) or 1640 medium (VivaCell, #C3010‐0500) supplemented with 10% fetal bovine serum (FBS) (Corning, #35‐179‐CV) and 1% penicillin–streptomycin antibiotic solution (Biosharp, #PYG0016).

### 2.8 Lentiviral Infection

According to the manufacturer's protocol, a cell suspension was prepared in complete medium (DMEM) at a density of 3–5 × 10^4^ cells/ml, with the specific cell density adjusted based on the size of the cultured cells. The cells were incubated at 37°C for 16–24 h until they reached 20%–30% confluence. Based on the cell MOI and viral titre, the appropriate volume of virus was added. The cells were incubated at 37°C for 16 h, after which the medium was replaced with complete medium for continued culture. (If there are changes in cell morphology, the medium can be changed earlier at around 8 h to maintain normal cell growth.) Approximately 72 h post‐infection, the cell condition was assessed under a microscope, and the medium was replaced to continue culture. After 48 h, the cells were selected with puromycin (2.00 μg/mL for A549 cells) for 3–4 days to establish stable BEST3 overexpression cells. The successful construction of BEST3 overexpression cells was confirmed by observing the fluorescence of the BEST3 cells under a fluorescence microscope (Nikon Eclipse Ti2, Japan).

### 2.9 Cell Counting Kit‐8 (CCK‐8) Assay

The proliferation ability of A549 cells was assessed using the Cell Counting Kit‐8 (CCK‐8) (Biosharp, #AR1160‐500). Logarithmically growing cells (1 × 10^3^ cells per 100 μL of medium per well) [21] were seeded into a 96‐well plate. At 0, 12, 24 and 48 h, 10 μL of CCK‐8 solution was added to each well, followed by incubation at 37°C in the dark for 2–4 h. The absorbance at 450 nm was measured using a microplate reader (Spectramax Absorbance Reader CMax Plus, Molecular Devices, USA) at the corresponding time points.

### 2.10 Cell Migration Assay

A549 cells (1 × 10^5^ cells) [21] were resuspended in 200 μL of serum‐free medium and seeded into the upper chamber of a Transwell system (Corning, #3422) with 8 μm pores, while the lower chamber was filled with 600 μL of medium containing 20% FBS. After 48 h, A549 cells were fixed with 4% paraformaldehyde for 20 min and stained with 0.1% crystal violet for 5–10 min. The upper chamber cells were removed with a cotton swab, and the cells on the membrane were counted and photographed using an inverted microscope. The number of migrated cells was quantified using ImageJ and recorded.

### 2.11 Wound‐Healing Assay

The apoptosis rate of A549 cells was detected using the YF647A‐Annexin V/PI apoptosis detection kit. After treatment with trypsin without EDTA, the cells were collected by centrifugation at 300 g for 5 min at 4°C. Following two washes with pre‐cold PBS, 100 μL of 1× Binding Buffer was added to 1–5 × 10^5^ cells. Subsequently, 5 μL of Annexin V‐APC and 5 μL of PI‐PE staining solution were added, and the cells were incubated in the dark at room temperature for 10–15 min. Finally, 400 μL of 1× Binding Buffer was added, mixed thoroughly and analysed by FCM.

### 2.12 Data Analysis and Statistical Methods

All statistics in this study were analysed with R software, and the R package chosen for data integration and plotting was tidyverse. Survival analysis curves were plotted with Kaplan–Meier, and log‐rank tests were applied to compare OS differences between groups. The following non‐parametric tests were used: Wilcoxon test and Kruskal‐Wallis test for comparison of continuous variables, Fisher exact test for comparison of unordered categorical variables, distribution of immunotherapy patients between different score groups, etc. Heat maps were drawn using the R package Complex Heatmap. Univariate and multivariate forest maps were drawn using the R package forest model. The experiments were independently repeated at least three times, and the data were analysed using ImageJ, R version 4.3.2 and GraphPad Prism. The numerical data followed a normal distribution, and variance was consistent between the groups undergoing statistical comparison. A reference value of *p*‐value < 0.05 was applied to specify that all analyses were statistically significant in this study. Statistical tests were uniformly labelled: *: *p* ≤ 0.05, **: *p* ≤ 0.01, ***: *p* ≤ 0.001, ****: *p* ≤ 0.0001.

## 3 Result

### 3.1 Acquisition of PRGDEs

**FIGURE 1:**
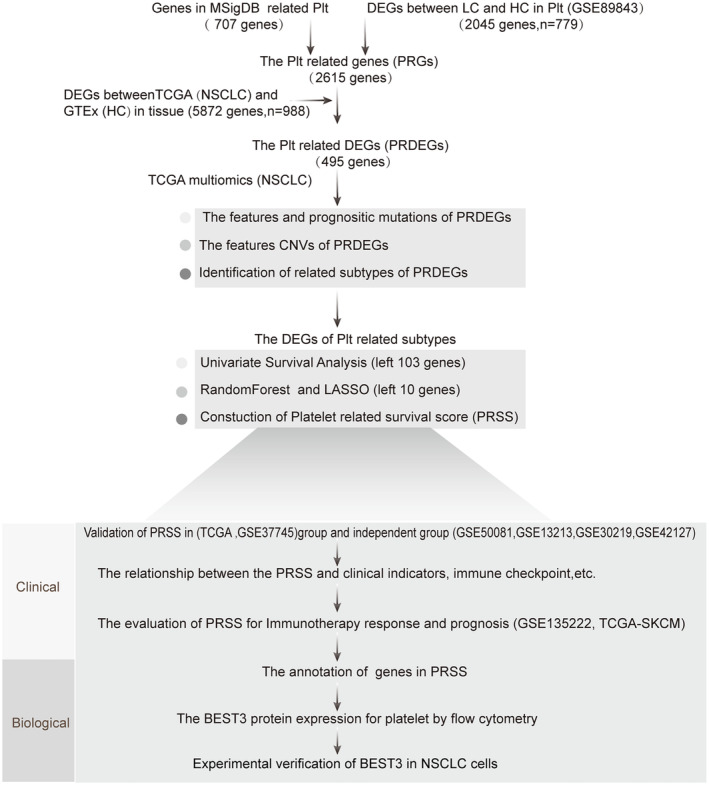
Flow chart of the study. The datasets required for this article were obtained from the Cancer Genome Atlas (TCGA) database, NCBI‐GEO database, MSigDB database, and the Genotype‐Tissue Expression (GTEx) database.

**FIGURE 2:**
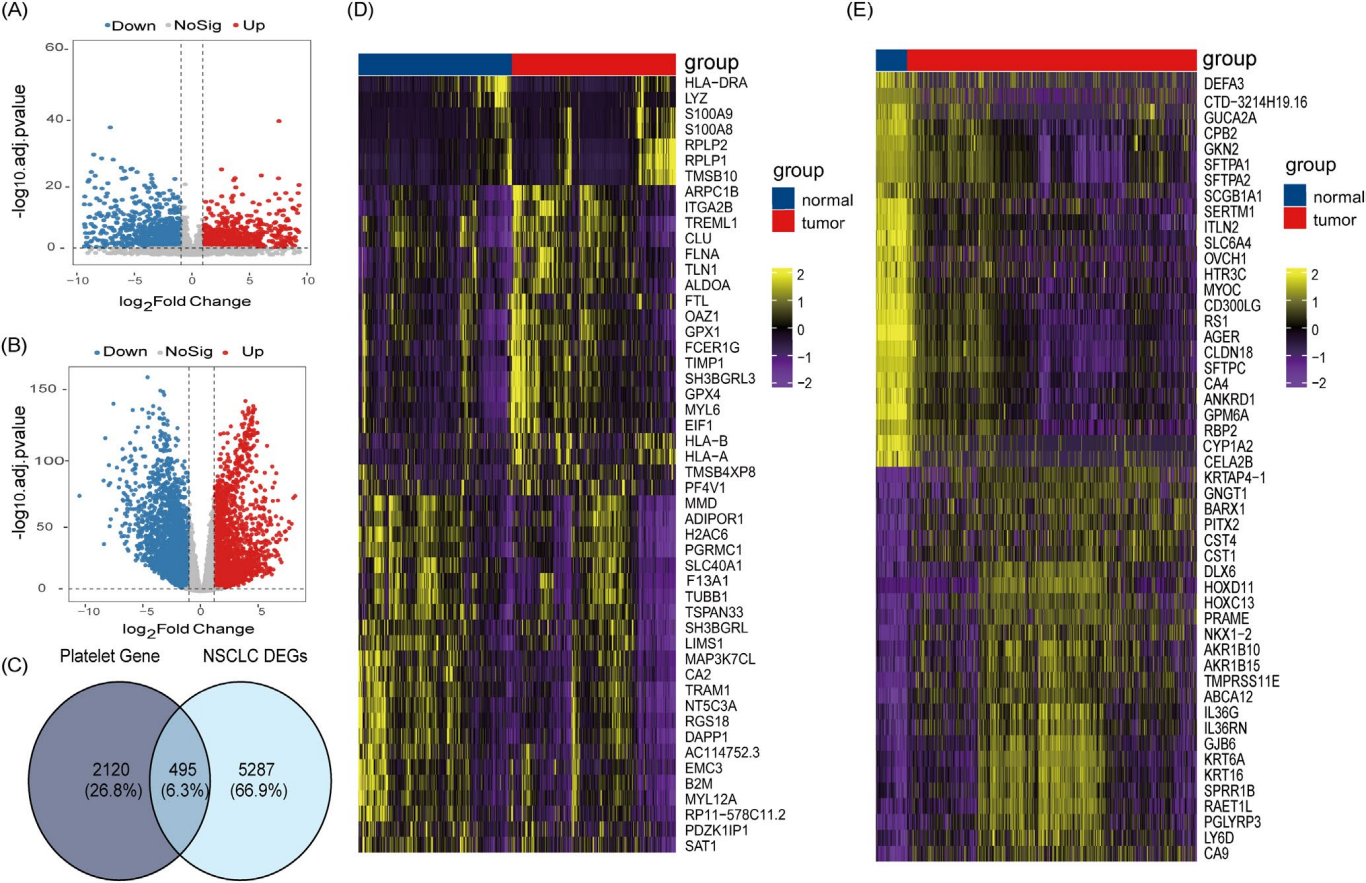
Acquisition of Platelet‐related Differentially Expressed Genes (PRDEGs). (A) Volcano plot of differential platelet genes in the GSE89843 dataset (log_2_|FC| > 1, adj.*p*.val < 0.01), blue indicates down‐regulated bases and red indicates up‐regulation. (B) Volcano plot of differential genes in the normal vs. disease group in the GTEx lung cancer dataset, (log_2_|FC| > 1, adj.*p*.val < 0.01), blue indicates down‐regulated base and red indicates up‐regulation. (C) PRDEGs were obtained by intersecting PRGs with NSCLC DEGs by Veen online mapping software. (D) Heatmap showing the 50 genes with significant differences. (E) The 50 genes with significant differences were selected for heat map display.

As depicted in the workflow diagram (Figure 1 and Figure S1), we obtained the GSE89843 dataset, consisting of 779 samples, encompassing healthy and lung cancer samples, from the GEO database. Differential gene expression analysis was performed using the R package Limma, with the threshold criteria set at FDR < 0.01 and |log_2_FC| > 1. This process led to the identification of 2045 genes, comprising 895 upregulated genes and 1150 downregulated genes (Figure 2A). Subsequently, we selected the top 50 genes with the most significant differences and generated a heatmap (Figure 2B). To gain deeper insights into the gene functionality associated with platelets, we conducted gene enrichment analysis for 37 GO and REACTOME pathways related to platelets. The results and relevant pathways are available in the Appendix S1 (Figure S2). Additionally, we combined these 2045 genes with the 707 genes retrieved from the MSigDB database, which are associated with platelet pathways, resulting in a comprehensive set of 2615 PRGs. We then downloaded lung cancer adjacent normal tissue samples and NSCLC samples from the GTEx and TCGA databases, respectively. Differential analysis was carried out using the R package Limma, applying the criteria of FDR < 0.01 and |log_2_FC| > 1 as the standard. This analysis allowed us to identify DEGs specific to NSCLC (NSCLC DEGs), as shown in Figure 2C. Furthermore, we selected the top 50 genes with the most significant differences and visualised them in a heatmap (Figure 2D). Out of these, there were 2664 up‐regulated genes and 3118 down‐regulated genes. Finally, through an online Venn diagram analysis tool, we intersected the 2615 PRGs with NSCLC DEGs, resulting in 495 PRDEGs, as illustrated in Figure 2E.

### 3.2 Multi‐Omics Characterisation and Biological Analysis of PRDEGs

Following the identification of PRDEGs, we obtained somatic mutation data for LUAD and LUSC from the TCGA database. Mutated genes from the PRDEGs were filtered to create a waterfall plot, highlighting the top 20 genes with the most significant differences, as depicted in Figure S3A. Out of 1059 samples, 822 displayed mutations, with the TNN gene having a relatively high mutation frequency of about 58%. To assess whether PRDEGs can differentiate patients' prognostic outcomes, we plotted Kaplan–Meier (KM) curves in the training dataset, stratified by mutation status, focusing on the top 6 genes with the most significant differences, as shown in Figure S3B. Next, leveraging copy number variation (CNV) data from the TCGA database, we examined the CNV status of PRDEGs. We selected the top 20 genes for representation, as illustrated in Figure S3C. Additionally, we provided boxplots for four genes with significantly different expression levels among the top 20, as depicted in Figure S3D.

### 3.3 Identification of Platelet‐Related Subtypes

**FIGURE 3:**
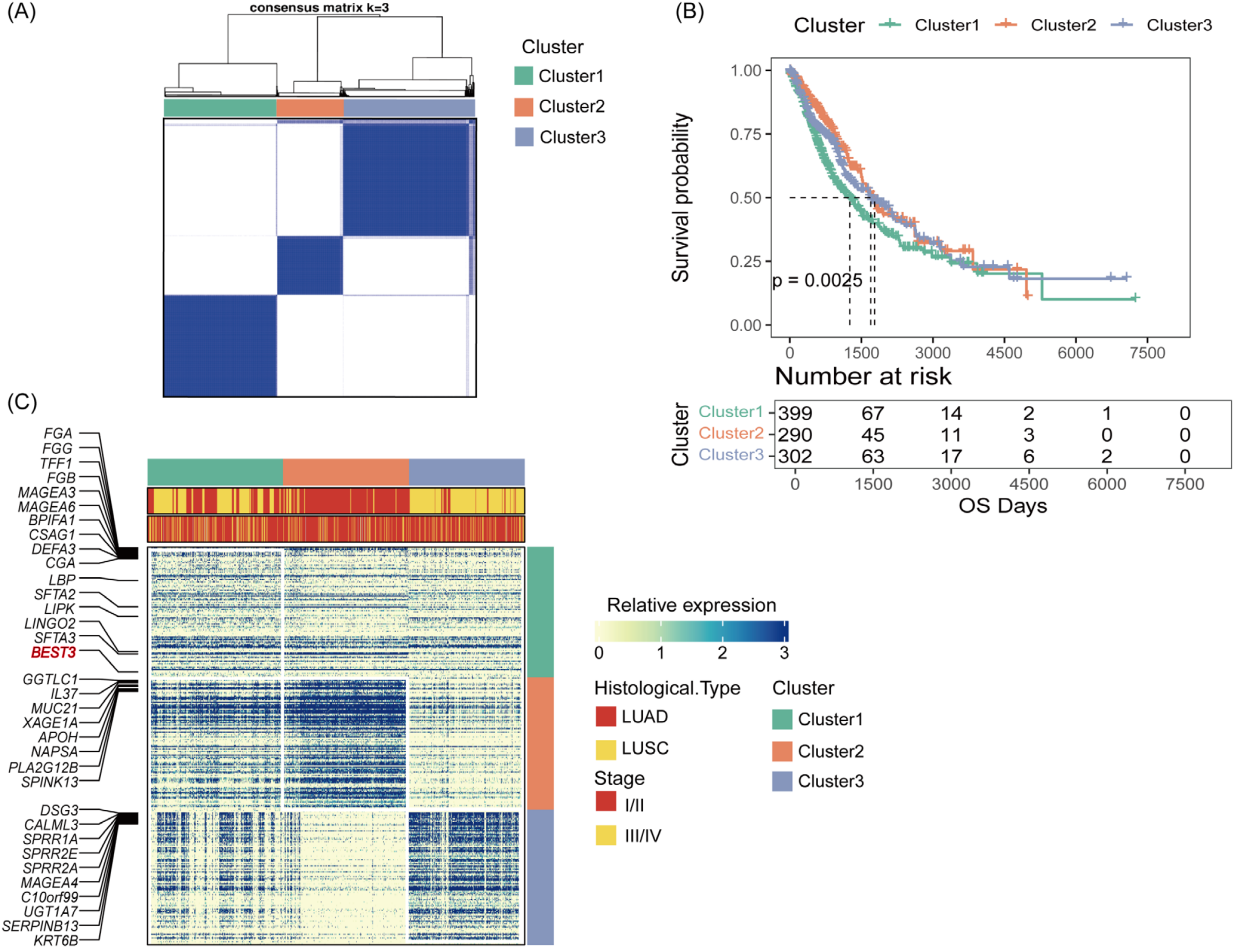
Identification of platelet‐related subtypes. (A) Typing of differential platelet‐associated genes, with the optimal number of clusters selected from three subtypes. (B) Differences between the subtypes of different cancer subgroups. Heatmaps demonstrating differential genes between the three subtypes, and selected TOP genes up‐regulated between each subtype were plotted in heatmaps for demonstration. (C) Comparison of survival prognosis among the three subtypes.

**FIGURE 4:**
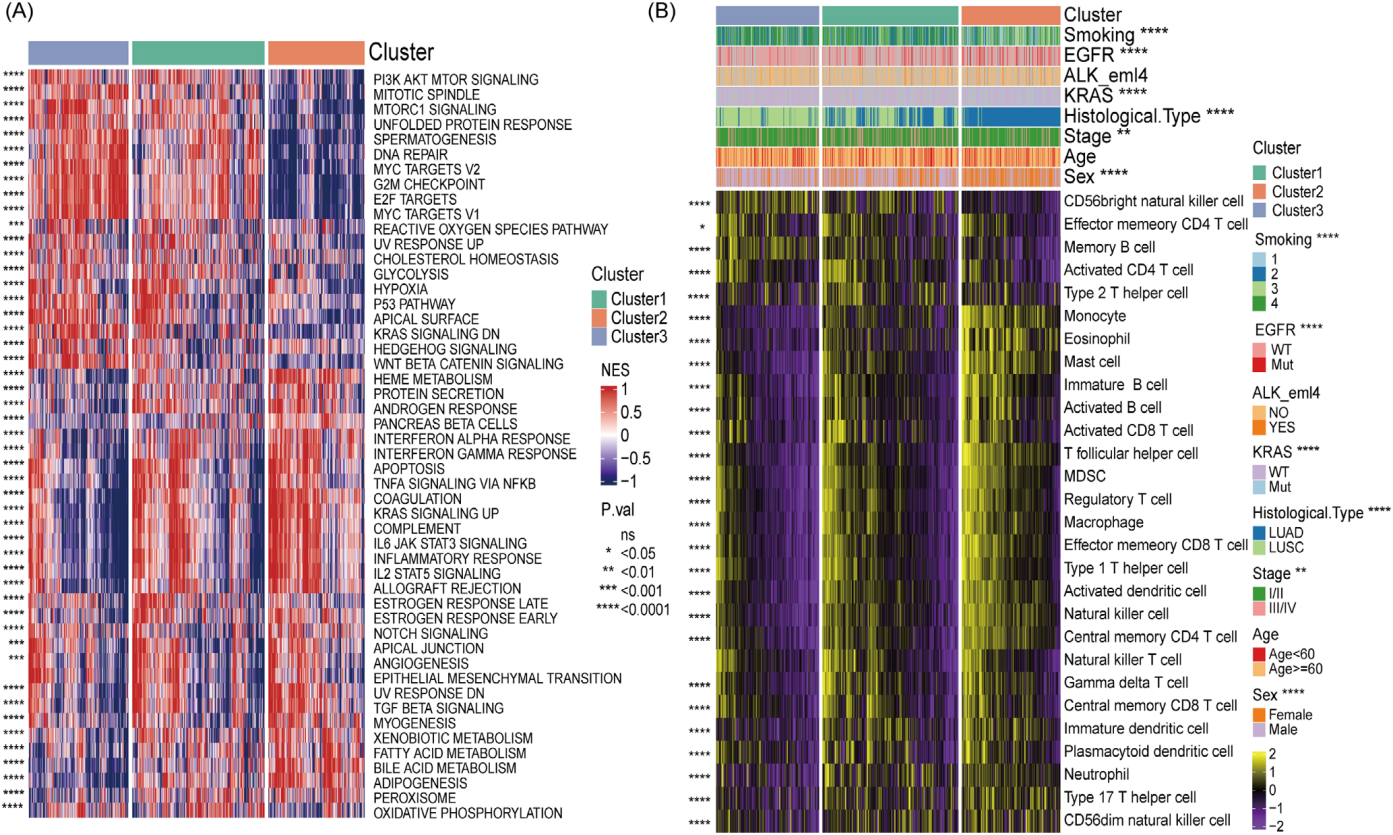
Comparison of differences between subtypes. (A) Comparison of differences in patient enrichment in the HALLMARK pathway between subtypes. (B) Comparison of immune infiltration of cells between subtypes. Statistical tests labelled: *p ≤ 0.05, **p ≤ 0.01, ***p ≤ 0.001, ****p ≤ 0.0001.

Using PRDEGs, we conducted a clustering analysis on a training dataset of NSCLC patients. We employed the Partitioning Around Medoids (PAM) clustering method with the Pearson correlation distance as the distance metric. After examining the Cumulative Distribution Function (CDF) descent curve, we determined that three clusters, with the most gradual descent, were the optimal number of clusters. This analysis successfully categorised patients into three distinct subtypes, as shown in Figure 3A. Furthermore, we observed significant differences in survival outcomes among these subtypes, with patients in Cluster 1 experiencing particularly unfavourable survival prognoses, as depicted in Figure 3B. To comprehensively understand the distinctions between these three subtypes regarding tissue composition and clinical staging, we obtained clinical data and expression matrices from the TCGA database for each subtype. R software was used to screen for the upregulated genes in each subtype, applying criteria of FDR < 0.01 and |log_2_FC| > 1. We then created a heatmap using the R package ComplexHeatmap, as shown in Figure 3C. Code reference for heatmaps [22]. The heatmap reveals that Cluster 3 subtype exhibits higher expression levels in LUSC, while the Cluster 2 subtype exhibits higher expression in LUAD. Notably, in the Cluster 1 subtype, both LUSC and LUAD genes are significantly expressed, with a majority of cases concentrated in stages III/IV. To further elucidate the differences between these subtypes and other tumour types, we retrieved clinical expression matrix information for LUAD and LUSC separately from the TCGA database. Using the R package ggplot2, we constructed a Sankey diagram to illustrate the distinctions between the three NSCLC subtypes, the two histological types (LUAD and LUSC) and the pathological staging, as presented in Figure S4. After determining different subtypes of platelet‐related genes, we compared the enrichment differences in HALLMARK pathways among different subtypes of NSCLC patients using the MSigDB database. Figure 4A illustrates significant enrichment of pathways related to cell growth, division, metabolism and stress response in Cluster1 and Cluster3 subtypes. These pathways and processes collectively regulate cell growth, division, survival, metabolism and stress response, with their abnormal activation or inhibition playing a crucial role in cancer and other diseases. In contrast, the Cluster2 subtype shows significant enrichment in pathways related to immune response, cell survival and death, inflammation and signal transduction. Additionally, we employed the ssGSEA method to compare the infiltration levels of immune cells among different subtypes. Figure 4B demonstrates that the Cluster1 subtype exhibits significant expression in most immune cell types, as well as in some specific functional cells such as regulatory and inhibitory cells. It also highlights the significant differences in immune cell types among the three subtypes.

### 3.4 Construction of the PRSS

**FIGURE 5:**
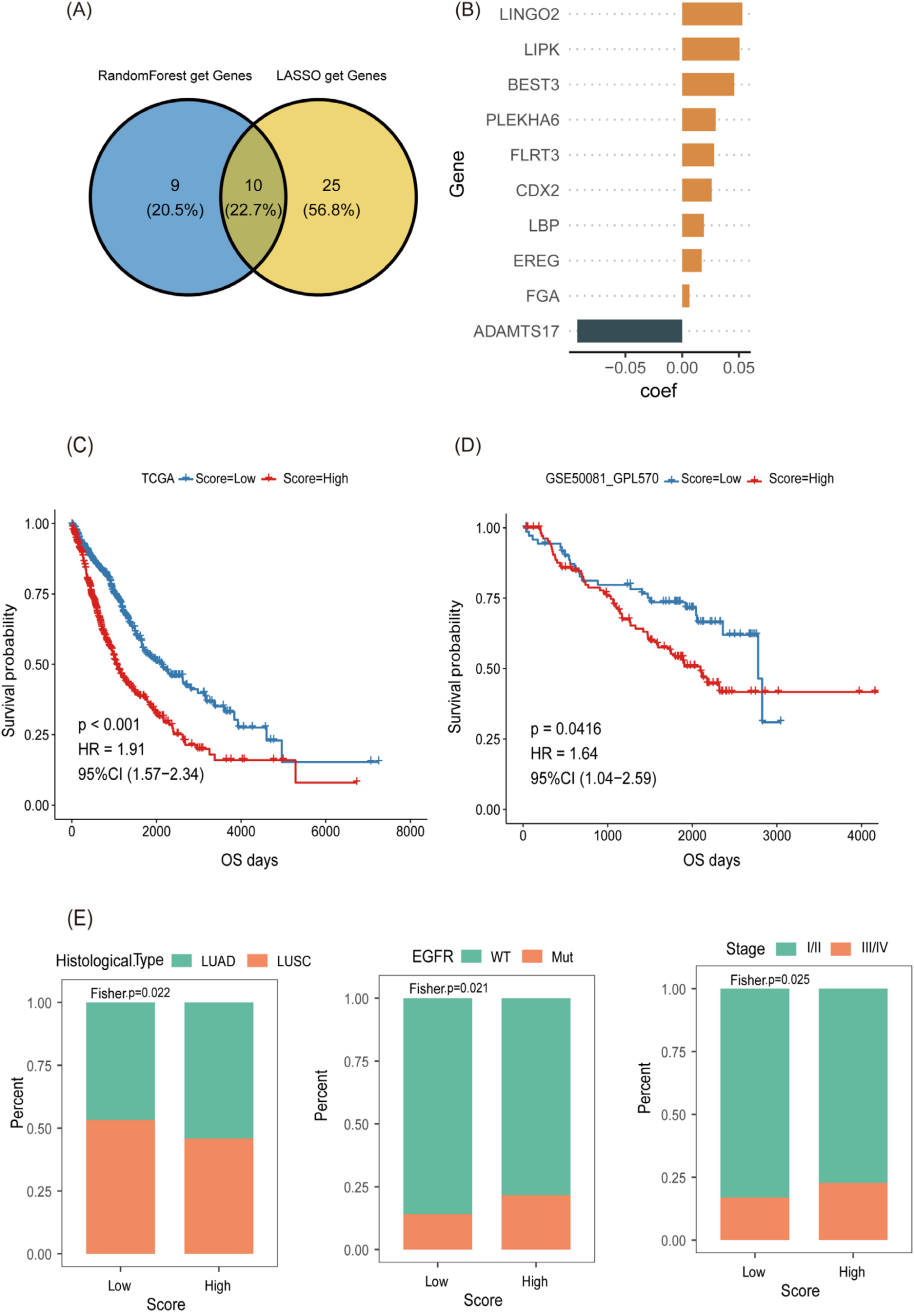
Construction and validation of the prognostic model PRSS. (A, B) Veen plots of model signature genes and bar graphs of corresponding coefficients were obtained. (C, D) Survival of patients in the high and low score groups in the training set cohort and the independent validation set cohort. (E) Association between model score and clinical characteristics of patients in the training set cohort.

After conducting a single‐factor COX regression analysis based on the expression profile of PRDEGs, we identified 103 genes that are significantly associated with prognosis (*p* < 0.01). Subsequently, we employed the Random Forest algorithm and the LASSO algorithm to select important features from these genes. In the Random Forest algorithm, we identified 19 feature genes, while in the LASSO‐COX algorithm, using the optimal lambda value, we selected 35 feature genes. By employing Venn diagram software, we identified 10 common prognosis‐related genes that were shared between these two methods, as shown in Figure 5A. Following this, we performed a multivariable COX regression analysis, calculating the regression coefficients for these feature genes. Notably, genes such as BEST3, LIPK and LINGO2 exhibited higher regression coefficients. Utilising this information, we constructed a prognostic model, and the distribution of coefficients for each feature gene is presented in Figure 5B. Following this, we performed a multivariable COX regression analysis, calculating the regression coefficients for these feature genes. Using the PRSS model, we computed a “Score” for each patient and stratified NSCLC patients into high and low Score groups. Kaplan–Meier survival curves, along with log‐rank tests, demonstrated a significantly reduced survival probability in the high Score group, as shown in Figure 5C. A similar analysis conducted in the independent validation dataset GSE50081 further confirmed that patients in the high Score group experienced significantly shorter survival times (Figure 5D). Additionally, we integrated another dataset, GSE37745, as a training set and employed GSE13213, GSE30129, and GSE42127 as validation datasets, all of which produced consistent findings. However, the survival differences in GSE42127 were not statistically significant (Figure S5). Following the construction of the prognostic model and its validation in the respective cohorts, we conducted a multivariable COX regression analysis to determine whether the Score independently predicts overall survival (OS). Even when accounting for other clinical factors, the multivariable COX analysis confirmed that the Score remained an independent predictive factor. Detailed results are provided in the Appendix S1 (Table S2A,B). Lastly, as depicted in Figure 5E, we conducted Fisher exact tests to explore the association between model scores (Score) and clinical characteristics of patients in the training cohort. The results revealed that the proportion of patients with LUAD in the low‐score group was lower than that in the high‐score group (Fisher *p* = 0.022). Moreover, in the analysis of the correlation between EGFR genotype and tumour stage, we observed that the proportion of EGFR mutation (Mut) patients and stage III/IV patients in the high‐score group was significantly higher than that in the low‐score group (Fisher *p* = 0.021 and *p* = 0.025, respectively). These findings indicate a significant correlation between model scores and these clinical features.

### 3.5 Immune Infiltration and Therapy

In order to better understand the association between score and immune characteristics, we calculated the immune infiltration of patients with high and low score groups in the training set cohort by the ssGSEA algorithm, as shown in the figure below (Figure S6A). From the figure, it can be seen that cells such as natural killer T cells and neutrophils are infiltrated to an increasing extent in the high‐scoring patient group. Meanwhile, we compared the expression of several major immune checkpoints between high and low‐scoring subgroups. From the figure, we could see that most immune checkpoints such as PDCD1 and CTLA4 had higher expression in the high‐scoring subgroup (Figure S6B). Then we retrieved the public immunotherapy dataset GSE135222 and analysed the response of different model subgroups to immunotherapy. In the NSCLC cohort (GSE135222‐GPL16791), the results were not significantly different, as shown in Figure S6C. In addition, we explored the other immunotherapy dataset TCGA‐SKCM and found that in the TCGA‐SKCM immunotherapy cohort, patients in the superior‐scoring subgroup had a significantly better survival prognosis than those in the low‐scoring subgroup and the immune response profile also showed an elevated expression. See (Figure S6D).

### 3.6 Validation and Annotation of BEST3 Gene

We conducted GO enrichment analysis on the ten feature genes (ADAMTS17, FGA, EREG, LBP, CDX2, FLRT3, PLEKHA6, BEST3, LIPK, LINGO2) identified in the previous section. The analysis revealed that the most significant processes were related to platelet function, including trauma response and cell differentiation. Notably, six out of the ten feature genes are known to participate in crucial platelet pathways, as shown in the Appendix (Figure S7A,B).

**FIGURE 6:**
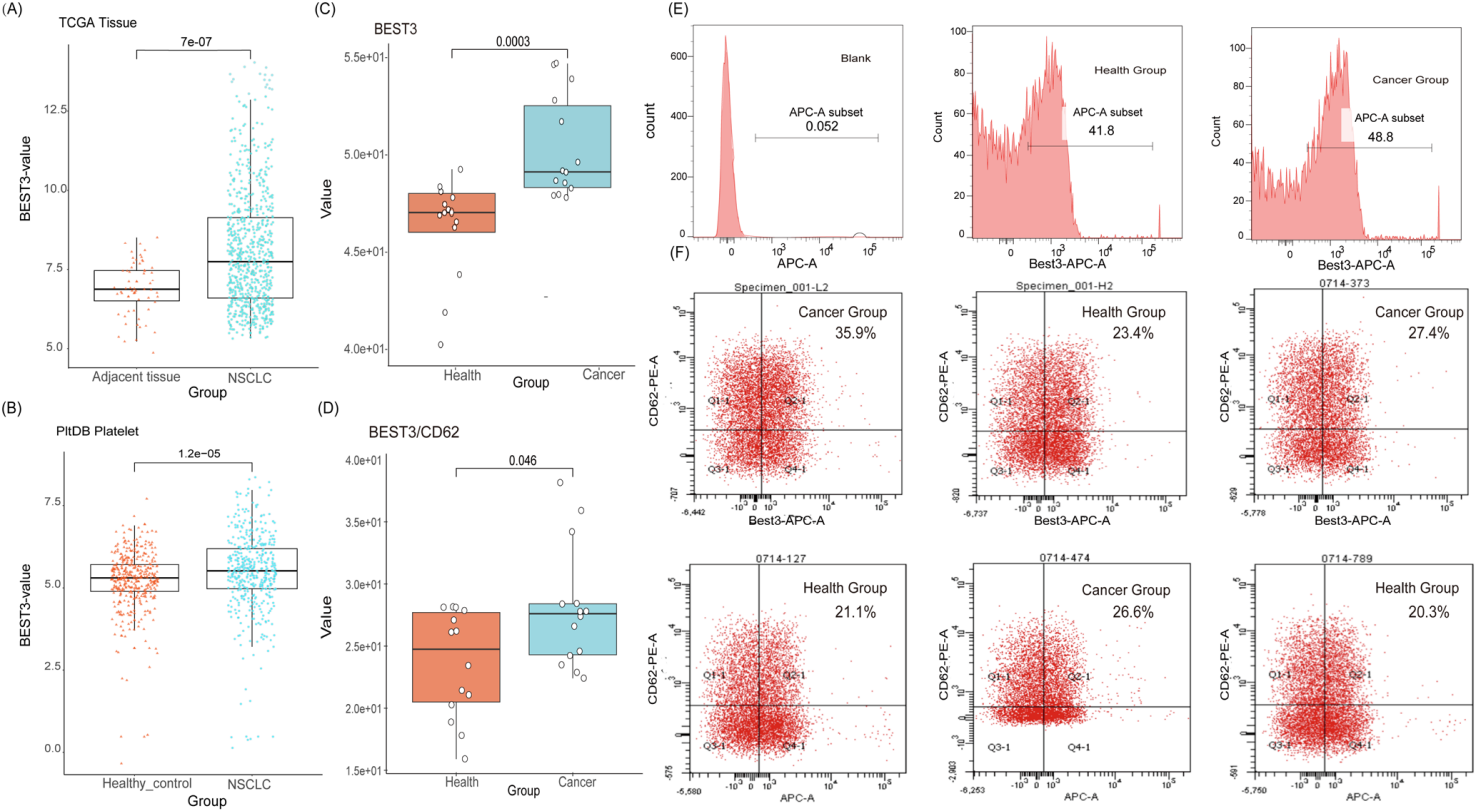
BEST3 Gene Expression and Protein Validation. (A) Differences between paraneoplastic and cancerous tissues, *p ≤ 0.05. (B) Difference in gene expression between the NSCLC and General Health groups, *p ≤ 0.05. Boxplots are based on quartiles, medians and third quartiles to illustrate the distribution of data. (C) Expression of BEST3 protein on platelets from tumour patients and healthy individuals. ***p ≤ 0.001. (D) Expression of BEST3 protein in activated platelets, *p ≤ 0.05. (E) FCM analysis of monostained BEST3 antibody. Plasma specimens were monostained with APC‐linked murine Bestrophin 3 antibody. In order, they are the negative control group, the healthy group, and the cancer group. (F) FCM analysis of activated platelets. Plasma specimens were double‐stained with PE‐coupled human CD62P antibody and APC‐coupled murine Bestrophin 3 antibody, respectively, with Bestrophin 3 APC‐A intensity on the horizontal axis and CD62P PE‐A intensity on the vertical axis.

Given the limited research on the expression of the BEST3 gene in platelets and its association with NSCLC, we conducted an in‐depth investigation into BEST3 gene expression and functionality. We found that BEST3 expression exhibited the most significant differences in Cluster1 within platelet‐related subtypes, as depicted in Figure 3B. Subsequently, we obtained expression data of the BEST3 gene from tissue samples using the UCSC Xena website and compared the expression levels between NSCLC tissue and adjacent non‐cancerous tissue using a bar chart. As shown in Figure 6A, we observed that the expression of the BEST3 gene in adjacent non‐cancerous tissue was significantly lower than in NSCLC tissue, with a *p*‐value of 7e−07, indicating a significant difference between the two groups.

We also analysed BEST3 gene expression between NSCLC patients and healthy individuals on the PltDB (Platelet Expression Atlas Database) website, presenting the results using boxplots. Notably, substantial differences in BEST3 gene expression were evident between cancer and healthy cohorts, as illustrated in Figure 6B. To understand BEST3 gene's protein‐level expression differences in platelets, we conducted FCM analysis, confirming BEST3 protein expression in both total and activated platelets. Selected FCM results are presented in Figure 6 (E: bar charts depicting BEST3 protein expression; F: scatter plots of BEST3 protein expression in activated platelets). Importantly, as shown in Figure 6C,D, our findings indicate that BEST3 protein expression is significantly higher in cancer patients compared to healthy individuals, whether in total platelets or activated platelets. Lastly, to explore the functional aspects of the BEST3 gene, we retrieved genes with similar profiles from the GEPIA website and conducted GO enrichment analysis. The results indicated that these functionally related genes were primarily associated with processes such as muscle cell differentiation and synaptic membrane structures, as detailed in Figure S5C. In addition, we investigated the relationship between BEST3 and several key immune checkpoint genes, including CD274, CD276, CD80, CD86, PDCD1 and CTLA4, in NSCLC patients using the UCSCXenaShiny platform (https://shiny.hiplot.cn/ucsc‐xena‐shiny/). Our analysis revealed statistically significant, though weak, correlations between the expression levels of BEST3 and each of the six immune checkpoint genes, with all *p*‐values < 0.05 (Figure S7D). Clinical information is provided in Table S4.

### 3.7 Prognostic Value and Functional Role of BEST3 in NSCLC

Based on the findings from Section 3.2, no significant SNPs were identified in the BEST3 gene in NSCLC. However, to eliminate the potential influence of other factors, we investigated somatic mutations and CNVs in the BEST3 gene using the CBioPortal platform (https://www.cbioportal.org/), as illustrated in Figure S1. Analysing TCGA data for LUSC and LUAD samples, we found relatively low mutation rates for BEST3 in NSCLC, specifically 1.63% in LUSC and 2.3% in LUAD (Figure S8B). These mutations are unlikely to have a significant impact on the gene's function, which suggests that variations in BEST3 expression may play a more critical role in patient outcomes. To further assess the prognostic significance of BEST3 expression in NSCLC, we analysed the TCGA dataset using the UCSCXenaShiny platform (https://shiny.hiplot.cn/ucsc‐xena‐shiny/). Patients were stratified into high and low BEST3 expression groups to evaluate survival outcomes. As shown in Figure S8C, high BEST3 expression was significantly associated with poorer survival in LUAD, while no significant difference in survival was observed in LUSC (*p* = 0.24, Figure S8D). This suggests that BEST3 expression may have a subtype‐specific prognostic impact, particularly in LUAD. Further supporting this notion, Cox regression analysis across pan‐cancer datasets revealed that elevated BEST3 expression was linked to poorer prognosis, especially in LUAD, where higher expression levels corresponded to significantly worse survival outcomes (Figure S8E). These findings indicate that BEST3 may serve as a critical prognostic marker in NSCLC.

**FIGURE 7:**
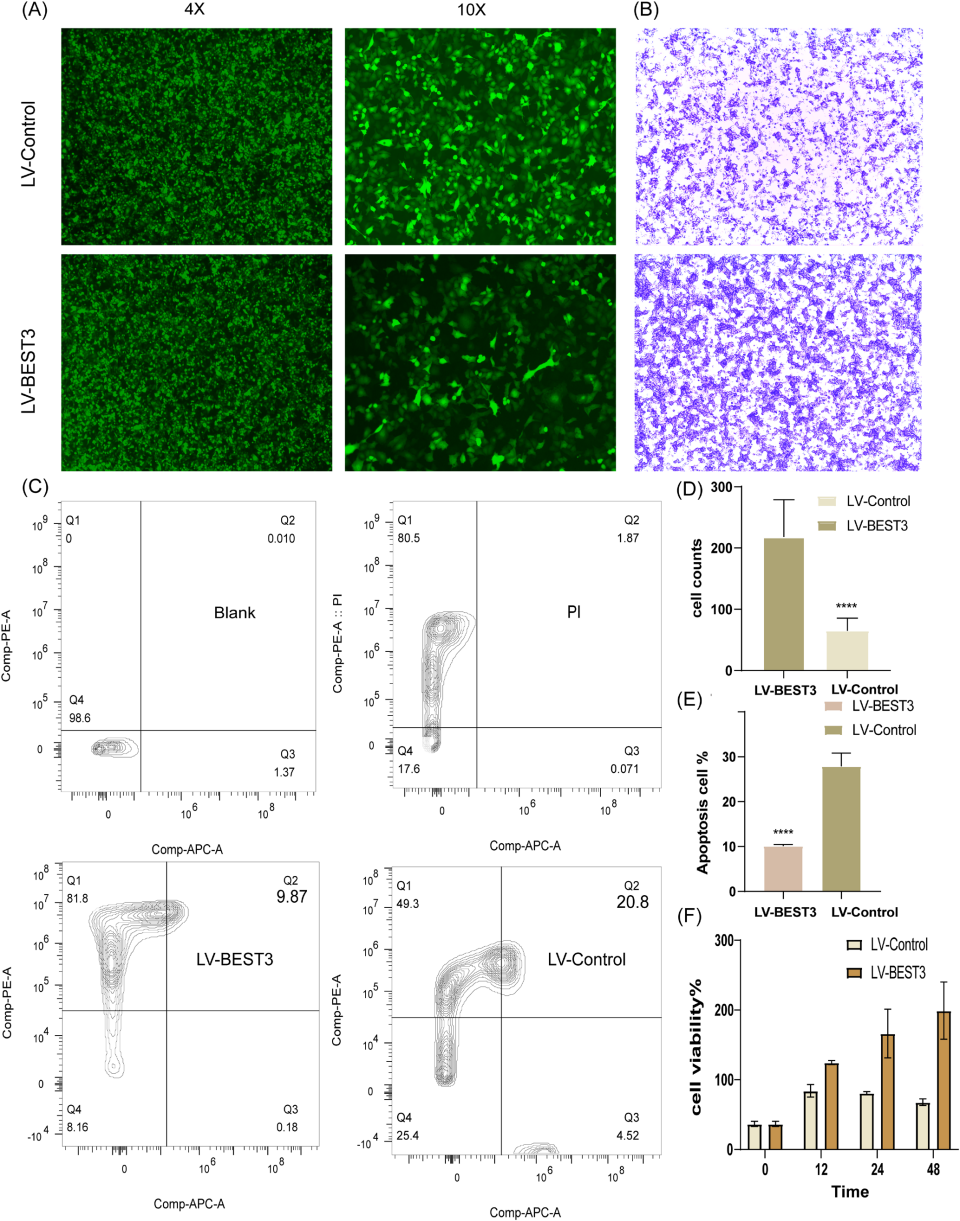
The function of BEST3 gene overexpression in NSCLC cells and validation of BEST3 protein expression. (A) Fluorescence microscopy images of LV‐BEST3 overexpressing cells and control LV‐Control cells. (B) Transwell migration assay showing the migratory ability of A549 cells with LV‐BEST3 overexpression compared to LV‐Control treated cells. (C) Flow cytometry analysis of apoptosis in LV‐BEST3 and LV‐Control cells. (D) Bar graph depicting cell migration capacity measured by Transwell assay (*p* < 0.005). (E) Bar graph of apoptosis rates (*p* < 0.005). (F) Bar graph of cell proliferation measured by CCK8 assay.

To explore the clinical implications of BEST3 expression more comprehensively, we examined its correlation with various clinical variables in NSCLC using the UALCAN platform. Our analysis revealed significant associations between BEST3 expression and several key clinical factors, including tumour stage, patient age, gender, smoking status, lymph node metastasis and TP53 mutation status. These results further underscore the potential of BEST3 as a valuable biomarker for predicting clinical outcomes in NSCLC (Figure S9). To experimentally validate the role of BEST3 in NSCLC, we generated BEST3‐overexpressing A549 cells. Fluorescence microscopy confirmed the successful transfection of LV‐BEST3 overexpression cells, as shown in Figure 7A. Functional assays revealed that BEST3 overexpression significantly enhanced the migration ability of A549 cells in the Transwell migration assay (Figure 7B,D). Additionally, the CCK‐8 cell viability assay demonstrated that BEST3 overexpression promoted the proliferation of A549 cells (Figure 7F). Furthermore, apoptosis analysis indicated that the apoptosis rate was reduced in BEST3‐overexpressing cells (Figure 7C,E).

In summary, both clinical and experimental data support the role of BEST3 as a key factor in NSCLC progression. BEST3 overexpression promotes tumour cell proliferation and migration while inhibiting apoptosis, highlighting its potential as a therapeutic target and prognostic biomarker in NSCLC.

## 4 Discussion

Despite extensive research on the role of platelets in NSCLC, the precise impact of platelet‐related subtypes remains unclear. In this study, we utilised clinical and genomic data to identify three distinct platelet subtypes: Cluster 1, Cluster 2 and Cluster 3, each demonstrating significant differences in tumour histology and clinical staging, highlighting the complexity of platelet involvement in NSCLC. Through differential expression analysis, we identified ten characteristic genes associated with NSCLC prognosis, with the BEST3 gene standing out as particularly significant. BEST3 is crucial in regulating NSCLC progression and metastasis, and its high expression correlates positively with patient survival. In vitro experiments confirmed its pivotal role in disease advancement. Our findings reveal abnormal BEST3 expression in cancer patients, validated through FCM and TCGA data, suggesting its potential as a clinical indicator for NSCLC progression and prognosis. This positions BEST3 as a promising target for novel therapeutic strategies in NSCLC.

The identification of 495 PRDEGs and the subsequent categorization into three distinct platelet subtypes underscore the pivotal role of these subtypes in NSCLC. Notably, the Cluster 1 subtype is closely associated with stage III/IV clinical staging, indicating a poorer prognosis. This suggests that different platelet subtypes may reflect distinct tumour characteristics and clinical outcomes, which could be crucial for patient stratification and personalised treatment approaches. Further analysis revealed that prognosis‐related genes, including BEST3, LIPK, LINGO2 and LBP, are predominantly present in the Cluster 1 subtype. This emphasises the poorer prognosis associated with this subtype and highlights the importance of these genes in NSCLC progression. Significant differences in PRSS prognostic model scores with respect to histological type, EGFR mutations and tumour staging further deepen our understanding of the intricate relationship between patient prognosis and disease characteristics.

Through TCGA data analysis, we observed a significant downregulation of BEST3 in adjacent non‐cancerous tissues compared to tumour tissues, indicating a potential role of BEST3 in NSCLC development and progression. The validation of BEST3 upregulation in the platelets of cancer patients using PltDB data, along with its substantial overexpression confirmed by FCM, points to its significance within the tumour microenvironment. These findings suggest that BEST3 may influence disease progression and patient prognosis through its involvement in platelet‐specific features in tumorigenesis. Additionally, GO enrichment analysis revealed the involvement of BEST3 in crucial biological processes such as myocyte differentiation, synaptic membrane contacts and cytoskeletal structure regulation. These insights provide a deeper understanding of the functional mechanisms of BEST3 in NSCLC and open up possibilities for its use as a therapeutic target or prognostic indicator. Future research focusing on the biological pathways associated with BEST3 could further elucidate its role in cancer biology and enhance its clinical applicability.

In addition, it has also been shown that BEST3 belongs to the Bestrophins family of chloride channels [23], which are thought to have cytoprotective functions and are critical in brain‐reactive astrocytes [24]. In addition, some studies have demonstrated that BEST3 plays a role in various cytoprotective mechanisms outside the brain. For example, Lei et al. [25] demonstrated that BEST3 protected basilar artery smooth muscle cells against hydrogen peroxide‐induced apoptosis; Guyla et al. [26] showed that BEST3 significantly ameliorated TNFα‐induced inflammation in endothelial cells; Wing‐Kee et al. [27] demonstrated that BEST3 expression reduced renal tubular epithelial cell cycle shortening caused by endoplasmic reticulum stress. Furthermore, significant research has shown that both classical Ca^2+^ activated Cl^−^ currents and cGMP^−^ dependent Ca^2+^ activated Cl^−^ currents have many features in common with heterologous Bestrophin expression. BEST3 was found to be associated with Ca^2+^ activated Cl^−^ currents, therefore confirming the notion that BEST genes encode Cl^−^ channels with significant biological functions [28]. There may be interference with the results due to the fact that Bestrophins can be made up of the same or different BEST subtypes [29]. Based on the results of the above research and our validation of the BEST3 gene, we can hypothesize that BEST3 may also function on platelets by Ca^2+^ activation and encoding Cl^−^ channels, but further research is needed to confirm this.

In summary, this study identified three platelet subtypes in NSCLC and highlighted the significant overexpression of the BEST3 gene in the Cluster1 subtype, offering new insights for prognostic assessment in NSCLC. However, the study has limitations, including a small sample size in FCM analysis and an incomplete understanding of BEST3's abnormal expression mechanisms. Future research should involve larger sample sizes and in‐depth molecular experiments to validate our findings and explore BEST3's functional mechanisms in NSCLC. These findings enhance our understanding of platelet diversity in NSCLC and open new avenues for personalised treatment strategies and prognostic assessment. BEST3, as a potential biomarker, may aid healthcare professionals in more accurate disease progression prediction and treatment planning. Continued research can further leverage this discovery to enhance medical care for NSCLC patients, ultimately improving survival rates and quality of life.

## Author Contributions

**Hanxiao Ren:** software (equal), validation (equal), visualization (equal), writing – original draft (equal). **Meng‐Ze Du:** conceptualization (equal). **Yulin Liao:** conceptualization (equal). **Ruiling Zu:** data curation (equal). **Lubei Rao:** data curation (equal). **Run Xiang:** resources (equal). **Xingmei Zhang:** resources (equal). **Shan Liu:** resources (equal). **Peiyin Zhang:** resources (equal). **Huaichao Luo:** data curation (equal), funding acquisition (equal), supervision (equal), writing – review and editing (equal). **Ping Leng:** supervision (equal). **Ling Qi:** supervision (equal).

## Ethics Statement

This study was approved by the Medical Committee of Sichuan Cancer Hospital (No. KY‐2021‐076). Due to the retrospective nature of this study, some survival data were only obtained through telephone follow‐ups because patients could not afford long journeys to reach the hospital. Only verbal informed consent was obtained from those patients or their legal guardians (for those passed away).

## Consent

The authors have nothing to report.

## Conflicts of Interest

The authors declare no conflicts of interest.

## Approval of the Manuscript

All authors have read and approved this manuscript.

## Supporting information


Appendix S1

## Data Availability

All data from study are included in this manuscript. Some of the code has been uploaded to GitHub (https://github.com/Huaichao2018/Platelet‐Related‐Subtypes‐Mining‐for‐NSCLC).
