# Supplementary material for: Deciphering the Significance of Platelet‐Derived Chloride Ion Channel Gene (BEST3) Through Platelet‐Related Subtypes Mining for Non‐Small Cell Lung Cancer
Source: J Cell Mol Med. 2024 Dec 21;28(24):e70233. doi: 10.1111/jcmm.70233 (PMC11662966; doi:10.1111/jcmm.70233)
Supplement: Supplementary file 1 — Appendix S1 [file JCMM-28-e70233-s001.zip › 3_Supplementary Material.pdf]

Supplemental Material

Deciphering the Significance of Platelet-Derived Chloride Ion Channel Gene (BEST3) through Platelet-Related Subtype Mining for Non-Small Cell Lung Cancer

Hanxiao Ren[1, 2*], Meng-Ze Du[3*], Yulin Liao[2*], Ruiling Zu[2], Lubei Rao[2], Run Xiang[4], Xingmei Zhang[1], Shan Liu[1], Peiyin Zhang[1], Ping Leng[1#] ,Ling Qi[5], Huaichao Luo[2#]

1. College of Medical Technology, Chengdu University of Traditional Chinese Medicine, Chengdu, Sichuan Province, People' s Republic of China.
2. Department of Clinical Laboratory, Sichuan Clinical Research Center for Cancer, Sichuan Cancer Hospital & Institute, Sichuan Cancer Center, Affiliated Cancer Hospital of the University of Electronic Science and Technology of China, Chengdu, China.
3. School of Health and Medical Technology, Chengdu Neusoft University, Chengdu, Sichuan Province, People' s Republic of China.
4. Department of Thoracic Surgery, Sichuan Cancer Hospital, Affiliate to the School of Medicine , The University of Electronic Science and Technology of China, Chengdu 610041, China.
5. Department of Core Medical Laboratory, the Sixth Affiliated Hospital of Guangzhou Medical University, Qingyuan People's Hospital

*These authors contributed equally to this work;
# Corresponding author
Correspondence:
Huaichao Luo, PhD, Department of Clinical Laboratory, Sichuan Cancer Hospital & Institute, Sichuan Cancer Center, School of Medicine, University of Electronic Science and Technology of China, No 55, Section 4, South People' s Road, Chengdu 610041, Sichuan, People' s Republic of China, Tel: +862885420344. Email: luo1987cc@163.com.
Ping Leng, College of Medical Technology, Chengdu University of Traditional Chinese Medicine , Chengdu 611137, China. Email: 596353806@qq.com.

SUPPLEMENTARY FIGURE LEGENDS

Supplementary Figure1. Flow chart of the study.

Supplementary Figure 2. GO enrichment analysis of the platelet-related gene pathway. Font size indicates the number of genes enriched in the pathway.

Supplementary Figure 3.Multi-omics characterization and biological analysis of differential platelet genes.(A) Differential platelet somatic mutations. (B) KM curves for the TOP6 mutation subgroup of the differential platelet gene; red indicates Mut mutations, blue indicates WT mutations; vertical coordinates indicate survival, horizontal coordinates indicate overall survival. (C) CNV alterations in differential platelet genes. (D) Differential expression of CNV-altered TOP4 genes.

Supplementary Figure 4. Identification of platelet-related subtypes.
The horizontal coordinates indicate the differences in grouping as well as pathological staging between LUAD, LUSC, and NSCLC tumors, and the vertical coordinates indicate how many. Differences in grouping between the three tumors were demonstrated by plotting Sankey diagrams.

Supplementary Figure 5. Training and validation of PRSS models. A.Training data set. B-D. Test data set.

Supplementary Figure 6.Immune infiltration and treatment.
(A) Immune infiltration in patients with high and low score groups. (B) Differential expression of major immune checkpoints.(C and D) Results of the analysis of immunotherapy in the GSE135222-GPL16791 cohort and TCGA-SKCM cohort of NSCLC.

Supplementary Figure 7.Validation and annotation of the BEST3 gene.
(A and C) GO enrichment analysis, the longer length of the bar graph indicates more genes enriched in the pathway. (B) Display of ten characterized genes associated with the platelet pathway.

Supplementary Figure 8.Somatic mutations,CNVs, and survival analysis of BEST3 in NSCLC .(A) Somatic mutations and CNVs were identified through the analysis of TCGA data samples from lung squamous cell carcinoma (LUSC) and lung adenocarcinoma (LUAD). (B) mRNA expression of BEST3 in CNVs.(C-D) Kaplan-Meier survival analysis of high and low BEST3 expression groups based on TCGA data. (E) The ratio of survival prognosis risk associated with BEST3 gene expression across pan-cancer analysis.

Supplementary Figure 9. Correlation between BEST3 expression and clinical indices in NSCLC.

Supplementary Table 1. Platelet-related pathway genes.

Supplementary Table 2. (A) Independent prognostic value of model score (TCGA training set cohort). **(B)** Independent prognostic value of model score (independent validation set cohort). **(C)** One-way cox analysis of training-focused models with other clinical factors. **(D)** validation of the centralized model with a one-way cox analysis of other clinical factors.

Supplementary Table 3. Univariate Analysis of Clinical Information Statistical Tables.

Supplementary Table 4. Clinical and characteristics of study patients from Sichuan Tumor H ospital.

Supplementary Figure 1

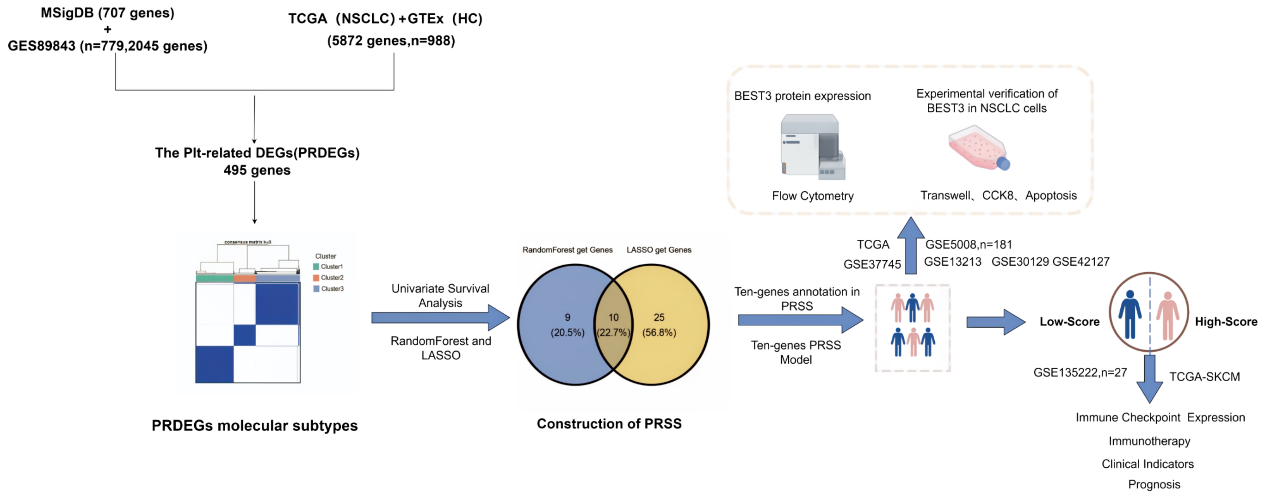

Supplementary Figure 2

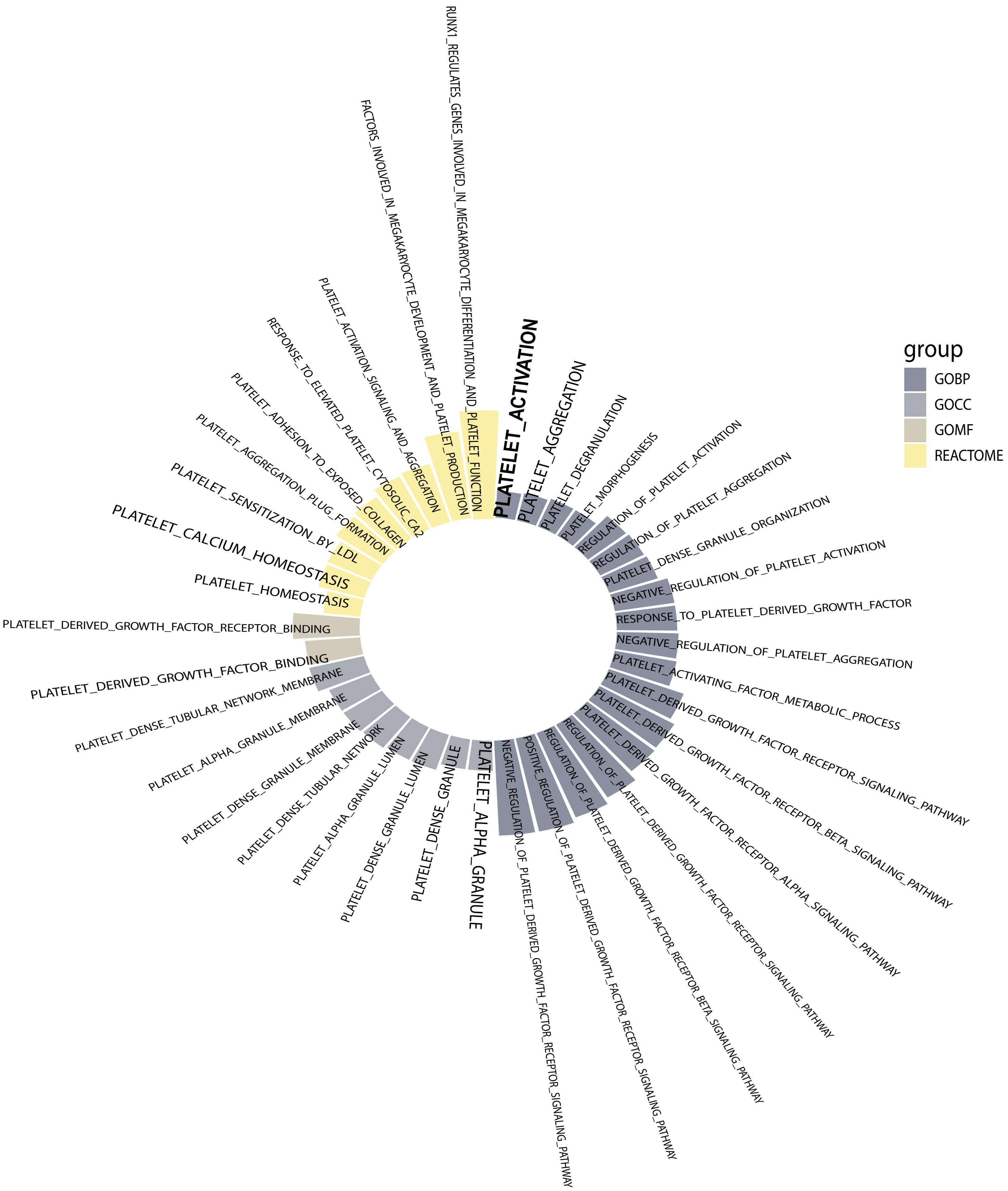

Supplementary Figure 3

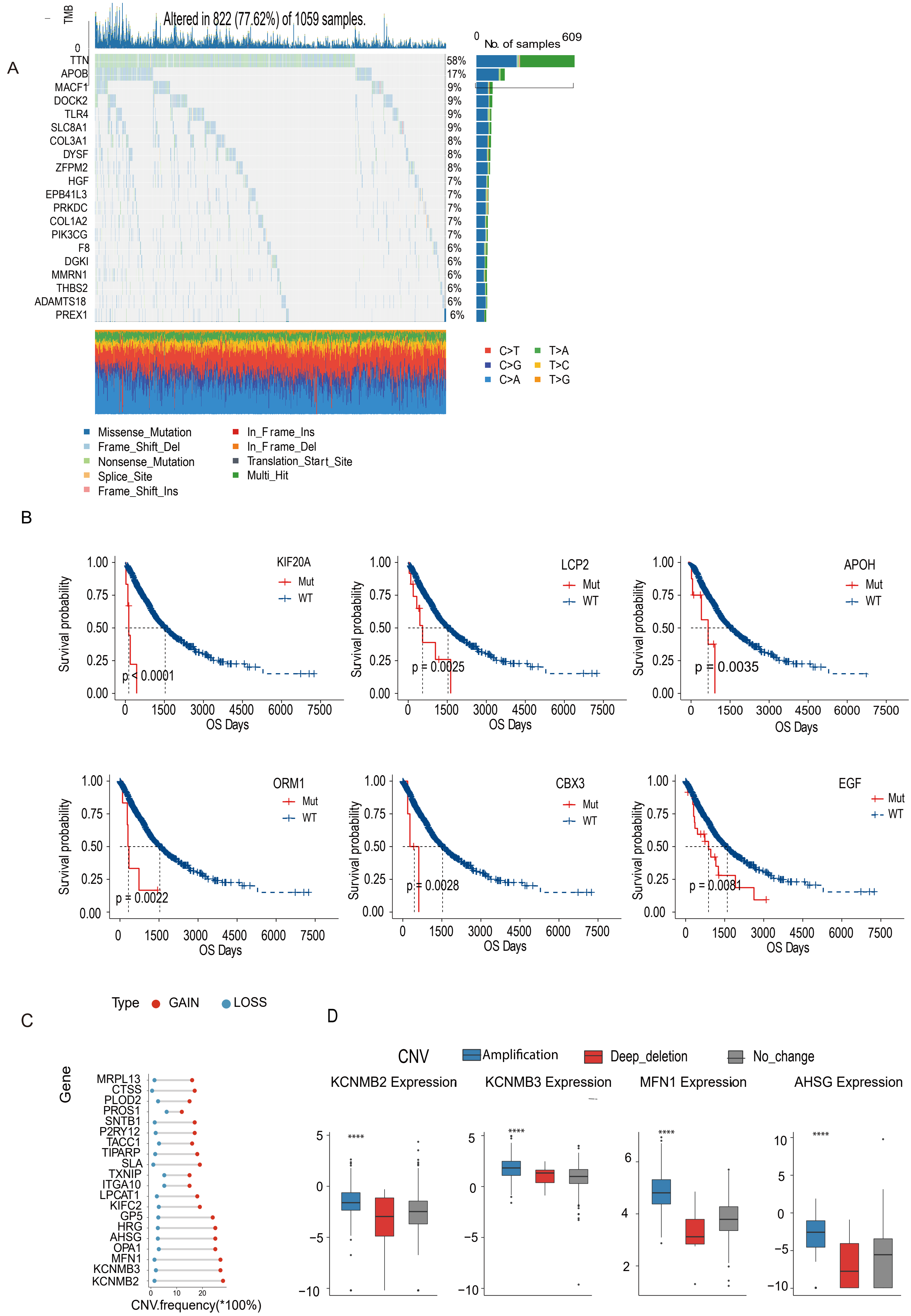

Supplementary Figure 4

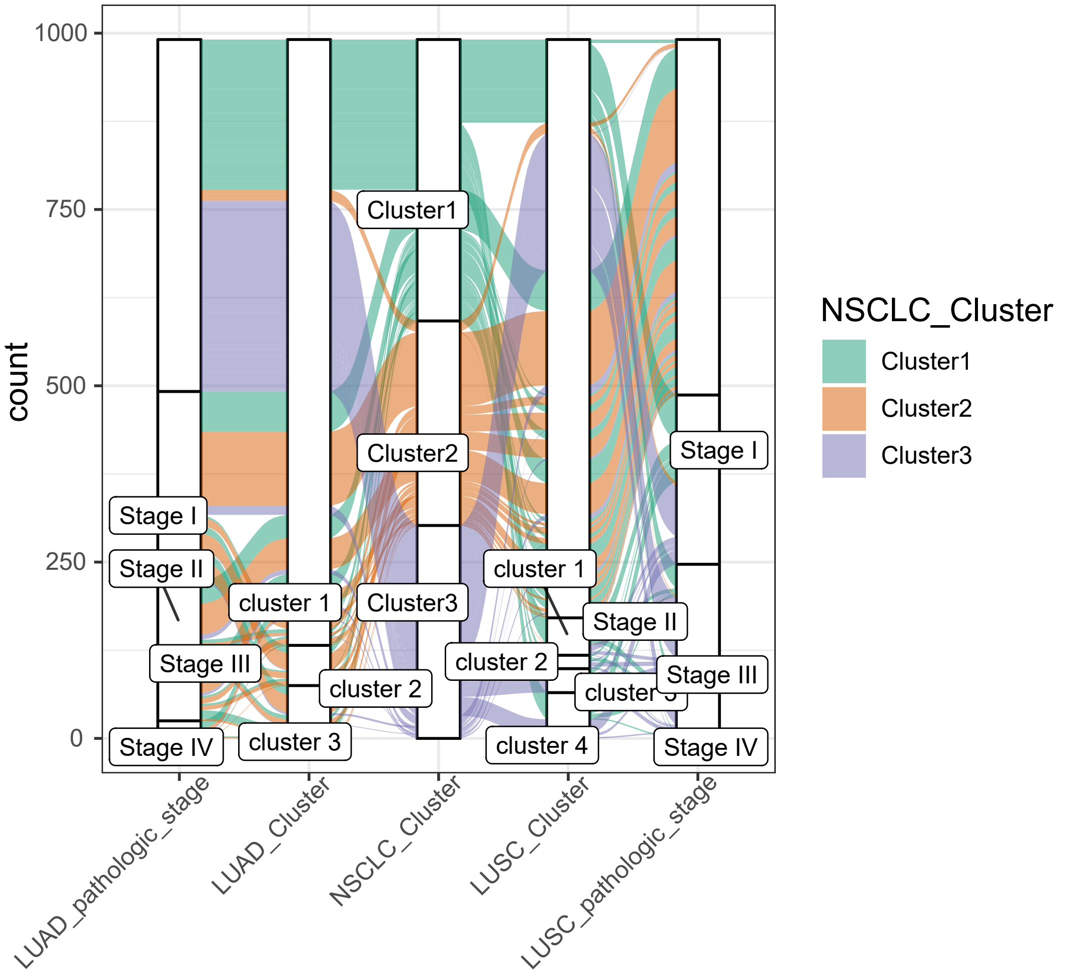

Supplementary Figure 5

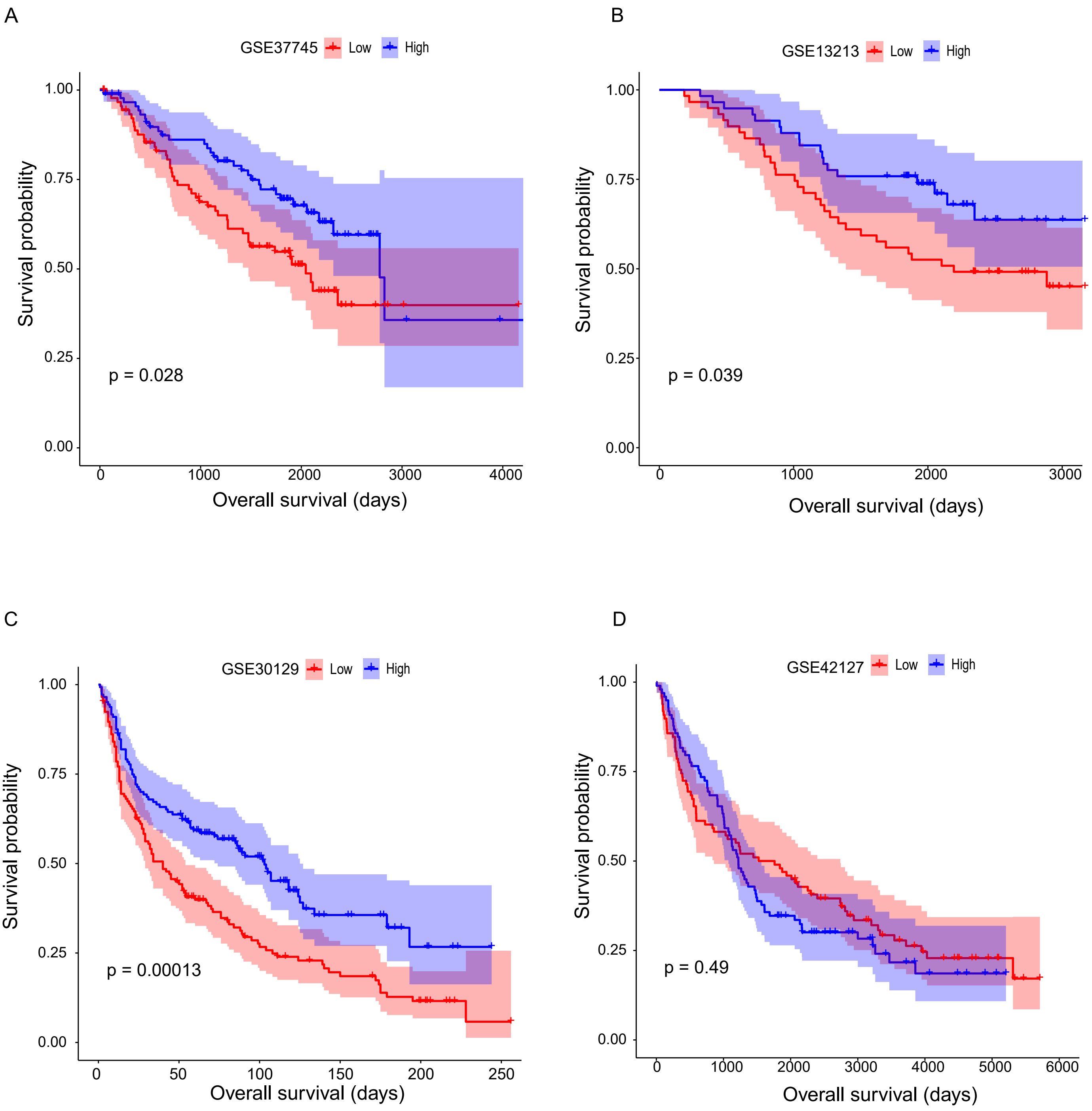

Supplementary Figure 6

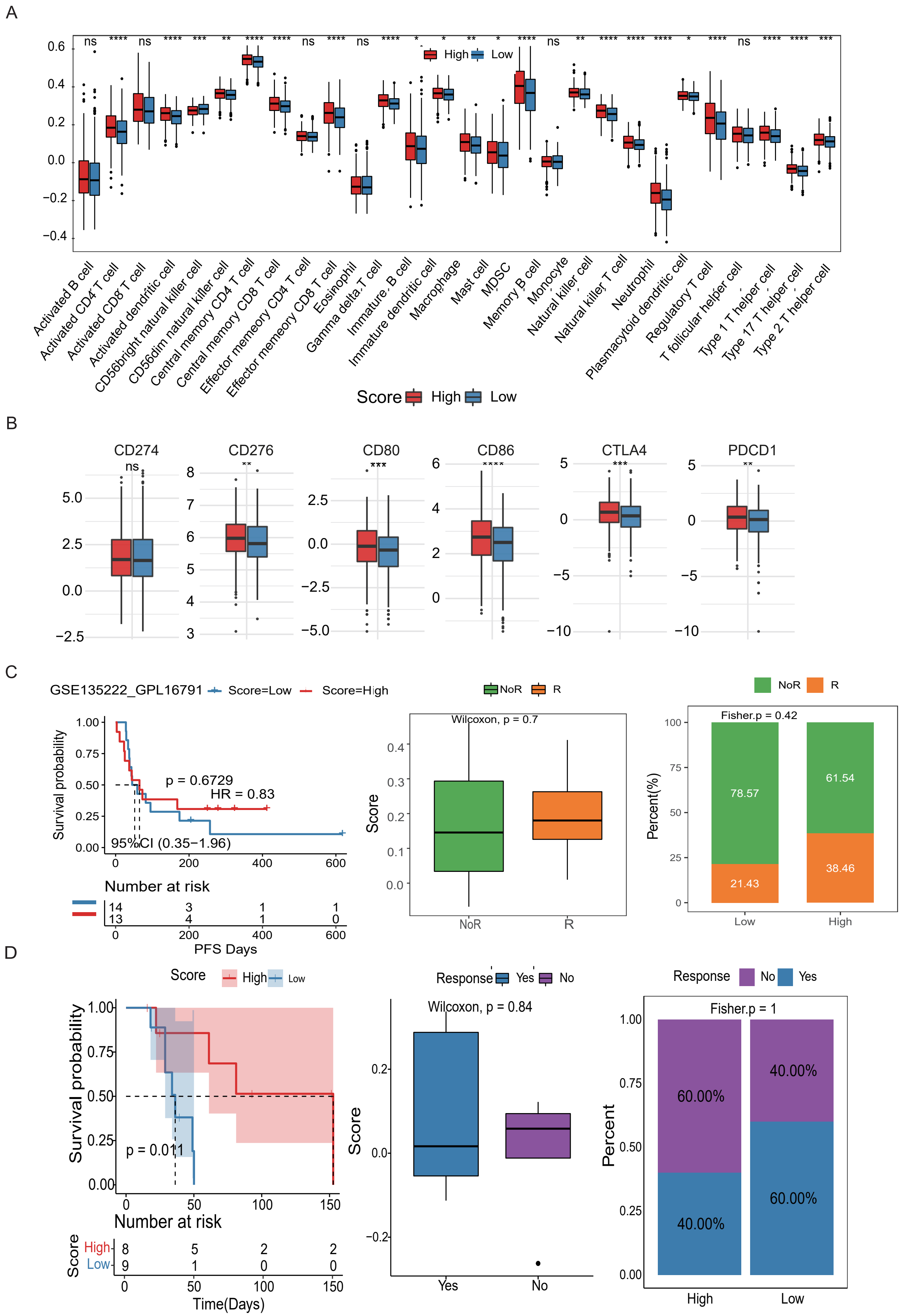

Supplementary Figure 7

A

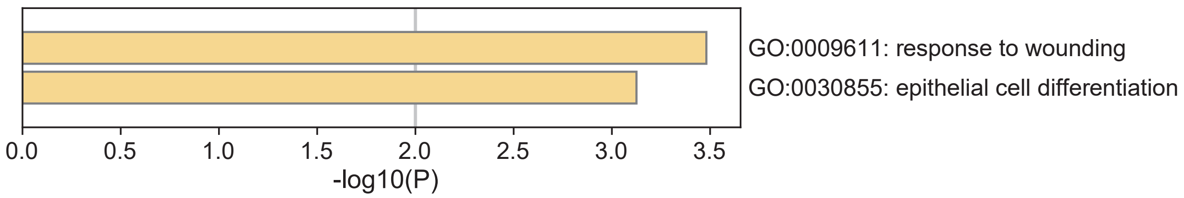

B

Genes	Platelet_related_Pathways
FGA	GOBP_PLATELET_DERIVED_GROWTH_FACTOR_RECEPTOR_ALPHA_SIGNALING_PATHWAY
LINGO2	REACTOME_PLATELET_AGGREGATION_PLUG_FORMATION
FLRT3	GOBP_REGULATION_OF_PLATELET_AGGREGATION
EREG	HP_IMPAIRED_COLLAGEN_INDUCED_PLATELET_AGGREGATION
LBP	HAY_BONE_MARROW_PLATELET
CDX2	HP_ABNORMAL_PLATELET_MEMBRANE_PROTEIN_EXPRESSION

C

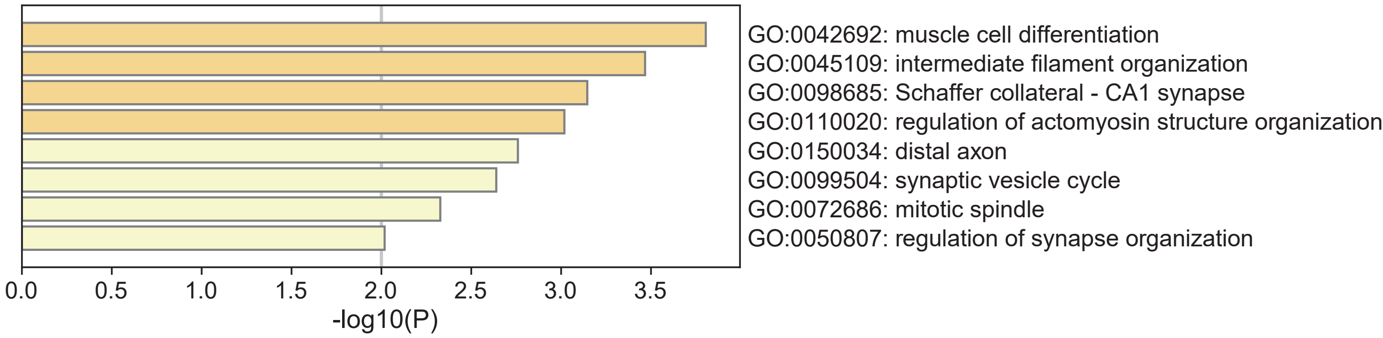

D

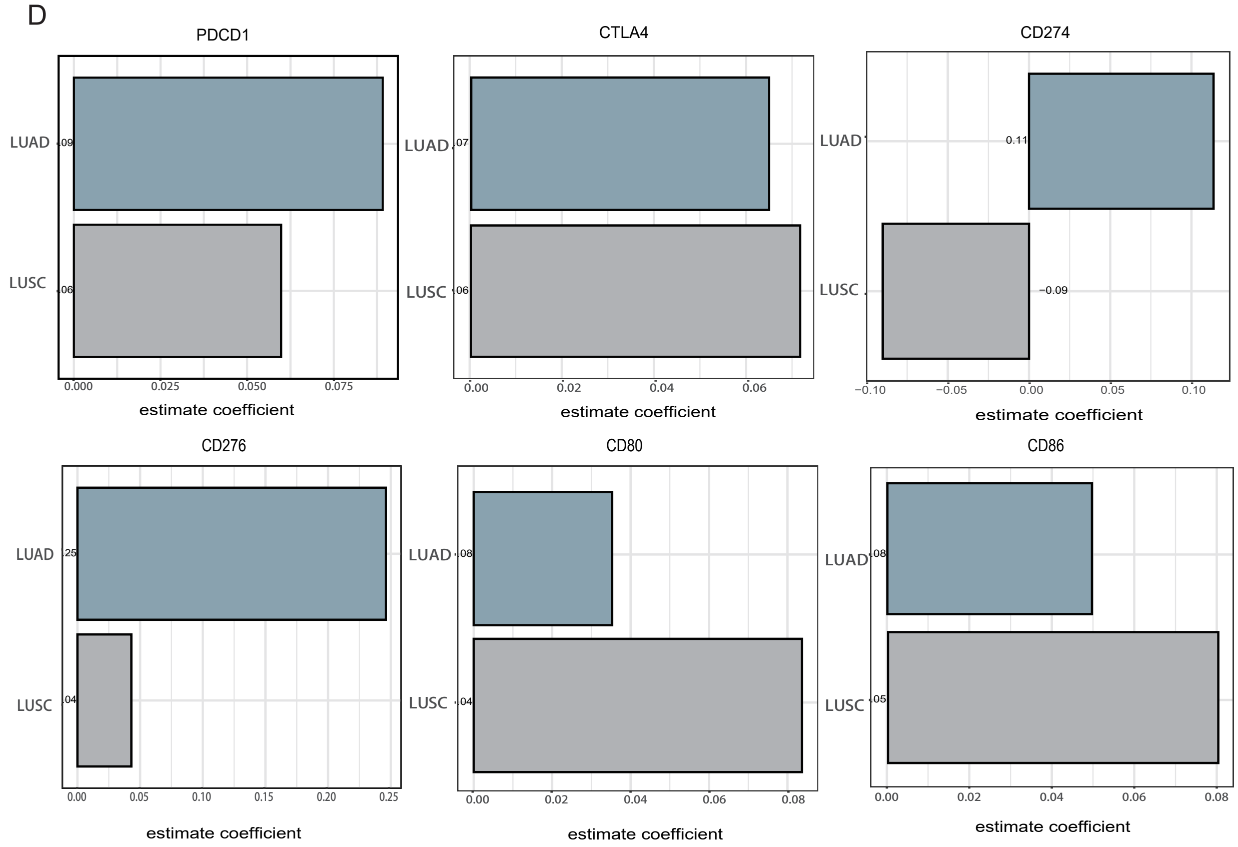

Supplementary Figure 8

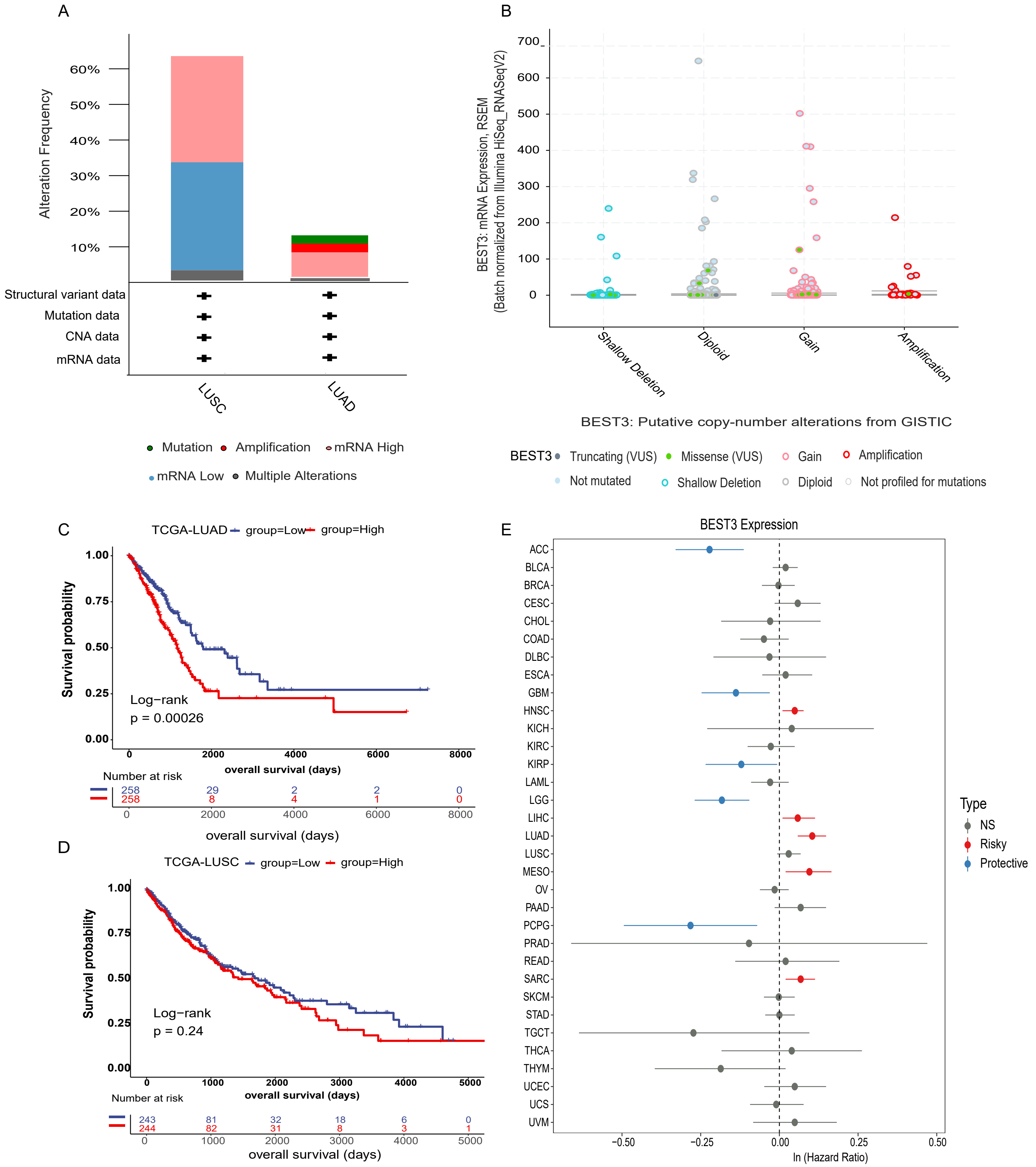

Supplementary Figure 9

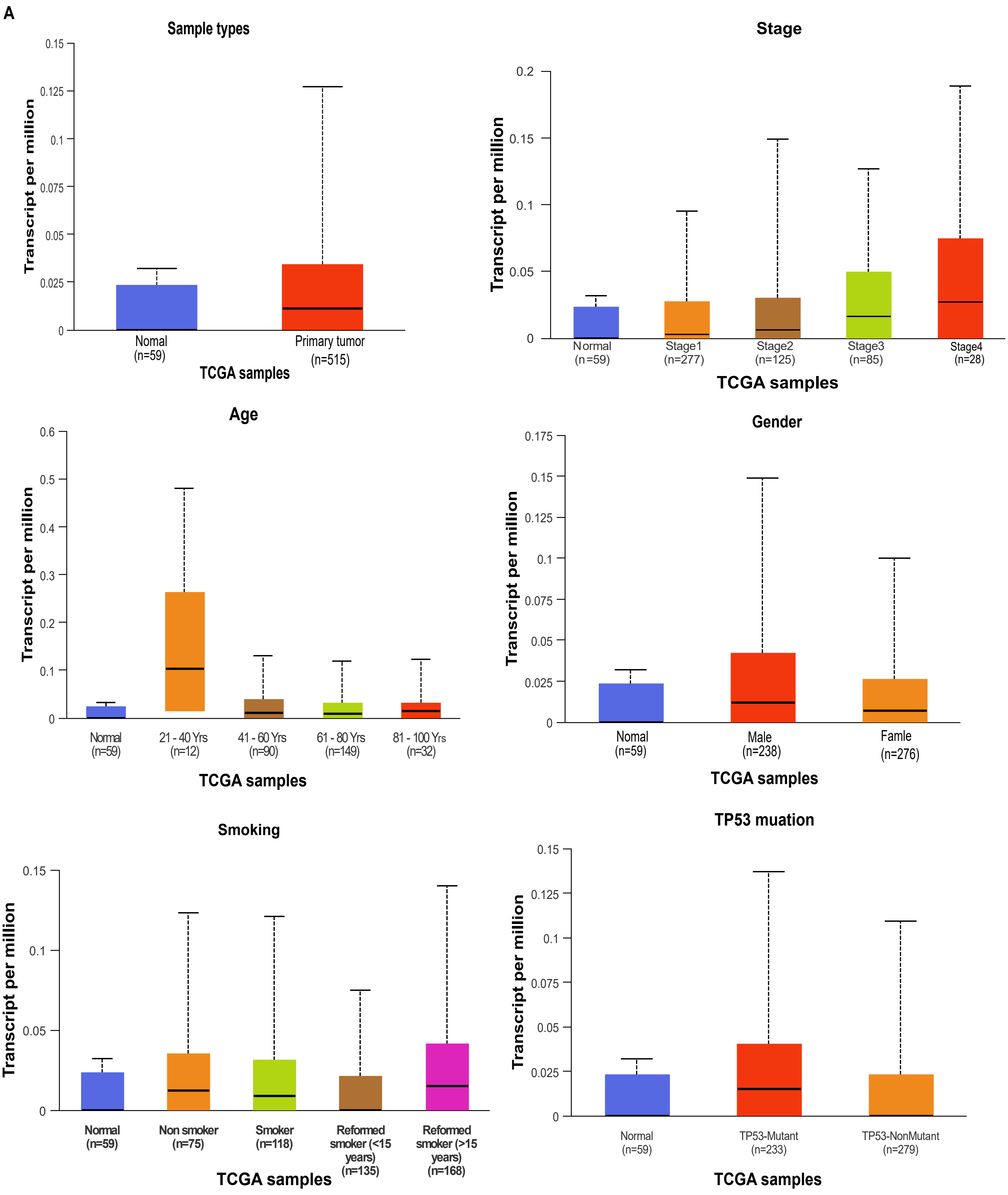

Supplementary Table 1

pathway
GOBP_PLATELET_DEGRANULATION
GOBP_REGULATION_OF_PLATELET_ACTIVATION
GOBP_NEGATIVE_REGULATION_OF_PLATELET_ACTIVATION
GOBP_REGULATION_OF_PLATELET_DERIVED_GROWTH_FACTOR_RECEPTOR_SIGNALING_PATHWAY
GOBP_POSITIVE_REGULATION_OF_PLATELET_DERIVED_GROWTH_FACTOR_RECEPTOR_SIGNALING_PATHWAY
GOBP_NEGATIVE_REGULATION_OF_PLATELET_DERIVED_GROWTH_FACTOR_RECEPTOR_SIGNALING_PATHWAY
GOBP_PLATELET_ACTIVATION
GOBP_PLATELET_DERIVED_GROWTH_FACTOR_RECEPTOR_ALPHA_SIGNALING_PATHWAY
GOBP_PLATELET_DERIVED_GROWTH_FACTOR_RECEPTOR_BETA_SIGNALING_PATHWAY
GOBP_RESPONSE_TO_PLATELET_DERIVED_GROWTH_FACTOR
GOBP_PLATELET_MORPHOGENESIS
GOBP_PLATELET_ACTIVATING_FACTOR_METABOLIC_PROCESS
GOBP_PLATELET_DERIVED_GROWTH_FACTOR_RECEPTOR_SIGNALING_PATHWAY
GOBP_PLATELET_DENSE_GRANULE_ORGANIZATION
GOBP_PLATELET_AGGREGATION
GOBP_REGULATION_OF_PLATELET_AGGREGATION
GOBP_NEGATIVE_REGULATION_OF_PLATELET_AGGREGATION
GOBP_REGULATION_OF_PLATELET_DERIVED_GROWTH_FACTOR_RECEPTOR_BETA_SIGNALING_PATHWAY
GOCC_PLATELET_DENSE_GRANULE_MEMBRANE
GOCC_PLATELET_DENSE_GRANULE_LUMEN
GOCC_PLATELET_ALPHA_GRANULE
GOCC_PLATELET_ALPHA_GRANULE_MEMBRANE
GOCC_PLATELET_ALPHA_GRANULE_LUMEN
GOCC_PLATELET_DENSE_TUBULAR_NETWORK
GOCC_PLATELET_DENSE_TUBULAR_NETWORK_MEMBRANE
GOCC_PLATELET_DENSE_GRANULE
GOMF_PLATELET_DERIVED_GROWTH_FACTOR_RECEPTOR_BINDING
GOMF_PLATELET_DERIVED_GROWTH_FACTOR_BINDING
REACTOME_PLATELET_HOMEOSTASIS
REACTOME_PLATELET_CALCIUM_HOMEOSTASIS
REACTOME_PLATELET_SENSITIZATION_BY_LDL
REACTOME_PLATELET_ADHESION_TO_EXPOSED_COLLAGEN
REACTOME_PLATELET_ACTIVATION_SIGNALING_AND_AGGREGATION
REACTOME_RESPONSE_TO_ELEVATED_PLATELET_CYTOSOLIC_CA2
REACTOME_PLATELET_AGGREGATION_PLUG_FORMATION
REACTOME_RUNX1_REGULATES_GENES_INVOLVED_IN_MEGAKARYOCYTE_DIFFERENTIATION_AND_PLATELET_FU
REACTOME_FACTORS_INVOLVED_IN_MEGAKARYOCYTE_DEVELOPMENT_AND_PLATELET_PRODUCTION

Supplementary Table 2

A

Variable		N	HR(95% CI)	p
**Score**	Low	258	Reference	
	High	232	2.59 (1.96 − 3.43)	<0.001
**Smoking**	No	51	Reference	
	Yes	439	1.10 (0.63 − 1.92)	0.725
**EGFR**	WT	421	Reference	
	Mut	69	0.95 (0.40 − 2.25)	0.903
**ALK_eml4**	NO	454	Reference	
	YES	36	1.17 (0.49 − 2.78)	0.721
**KRAS**	WT	434	Reference	
	Mut	56	1.02 (0.45 − 2.32)	0.970
**Histological.Type**	LUAD	228	Reference	
	LUSC	262	0.96 (0.71 − 1.30)	0.806
**Stage**	I/II	397	Reference	
	III/IV	93	1.82 (1.34 − 2.45)	<0.001
**Age**	Age<60	106	Reference	
	Age>=60	384	1.15 (0.81 − 1.63)	0.439
**Sex**	Female	208	Reference	
	Male	282	1.29 (0.96 − 1.73)	0.095

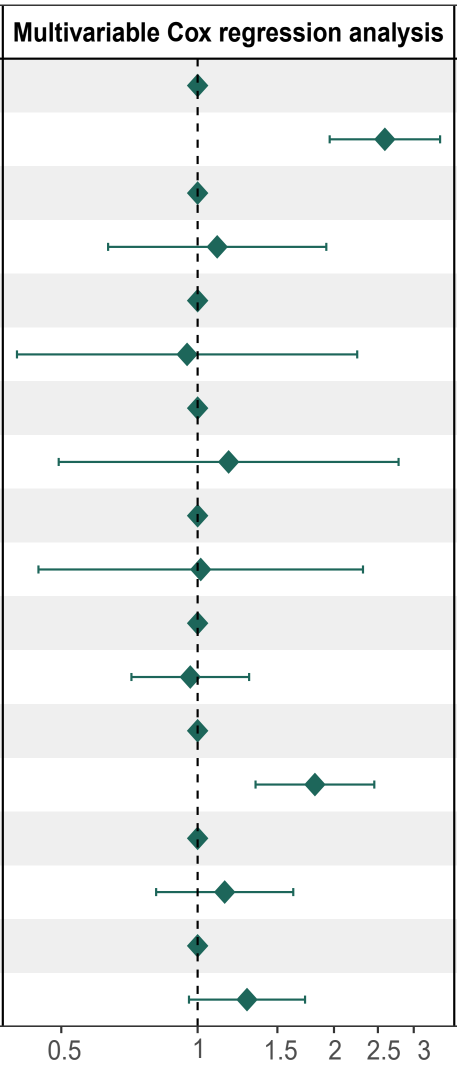

B

Variable		N	HR(95% CI)	p
**Score**	Low	66	Reference	
	High	94	1.74 (1.03 − 2.92)	0.037
**Smoking**	No	24	Reference	
	Yes	136	1.24 (0.57 − 2.66)	0.588
**Sex**	Female	72	Reference	
	Male	88	1.73 (1.01 − 2.94)	0.045
**Age**	<60	24	Reference	
	>=60	136	1.03 (0.51 − 2.11)	0.927

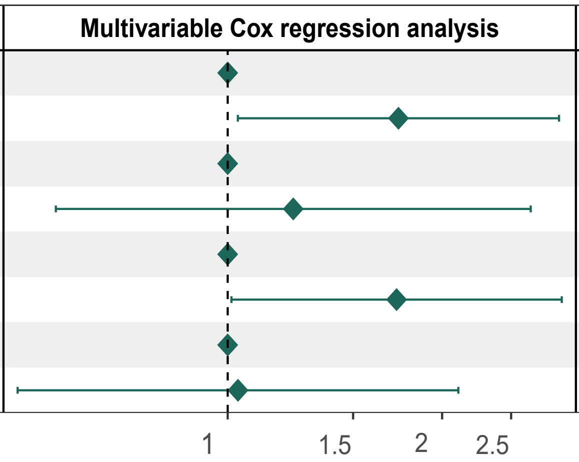

C

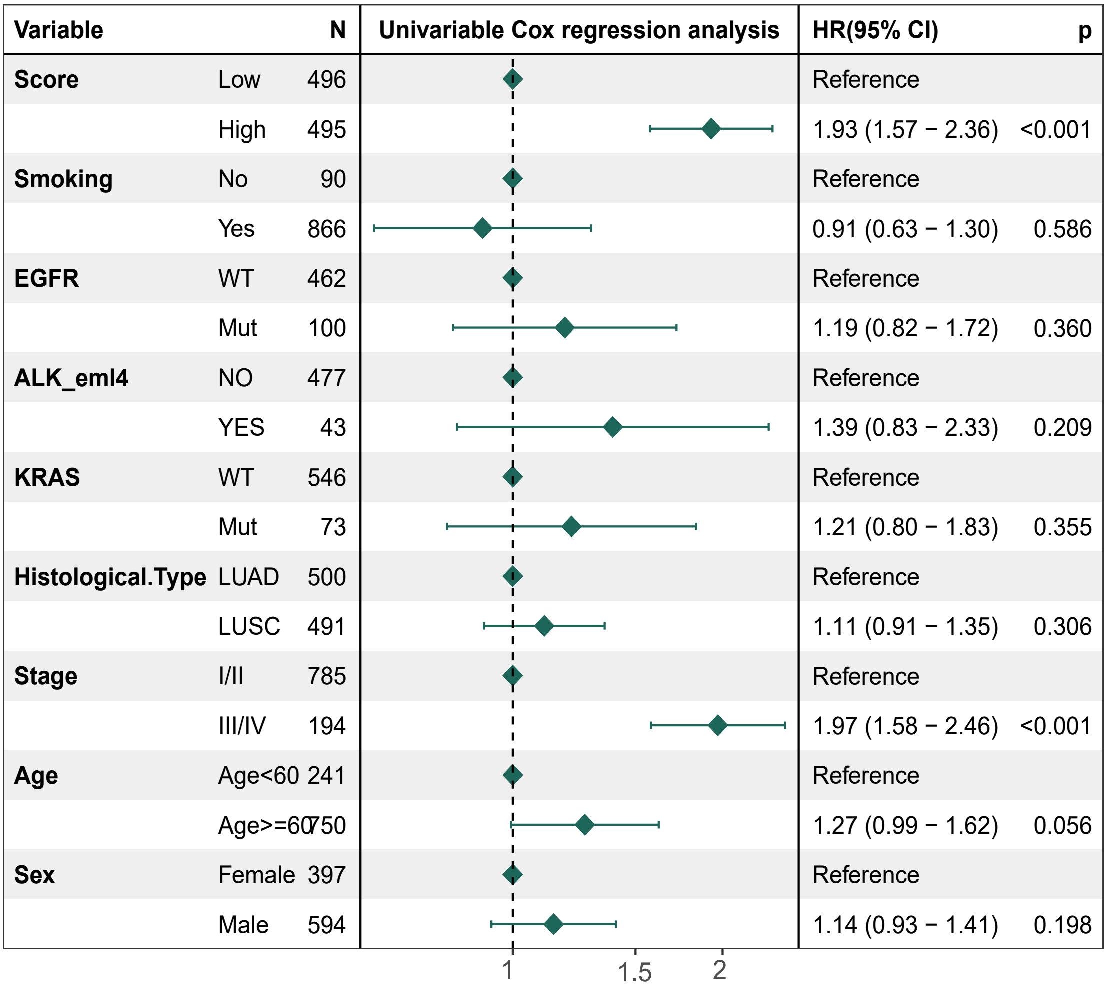
Variable N
Univariable Cox regression analysis
HR(95% CI) p
Score Low 496 Reference
High 495 1.93 (1.57 − 2.36) <0.001
Smoking No 90 Reference
Yes 866 0.91 (0.63 − 1.30) 0.586
EGFR WT 462 Reference
Mut 100 1.19 (0.82 − 1.72) 0.360
ALK_eml4 NO 477 Reference
YES 43 1.39 (0.83 − 2.33) 0.209
KRAS WT 546 Reference
Mut 73 1.21 (0.80 − 1.83) 0.355
Histological.Type LUAD 500 Reference
LUSC 491 1.11 (0.91 − 1.35) 0.306
Stage I/II 785 Reference
III/IV 194 1.97 (1.58 − 2.46) <0.001
Age Age<60 241 Reference
Age>=60 750 1.27 (0.99 − 1.62) 0.056
Sex Female 397 Reference
Male 594 1.14 (0.93 − 1.41) 0.198
1
1.5
2

D

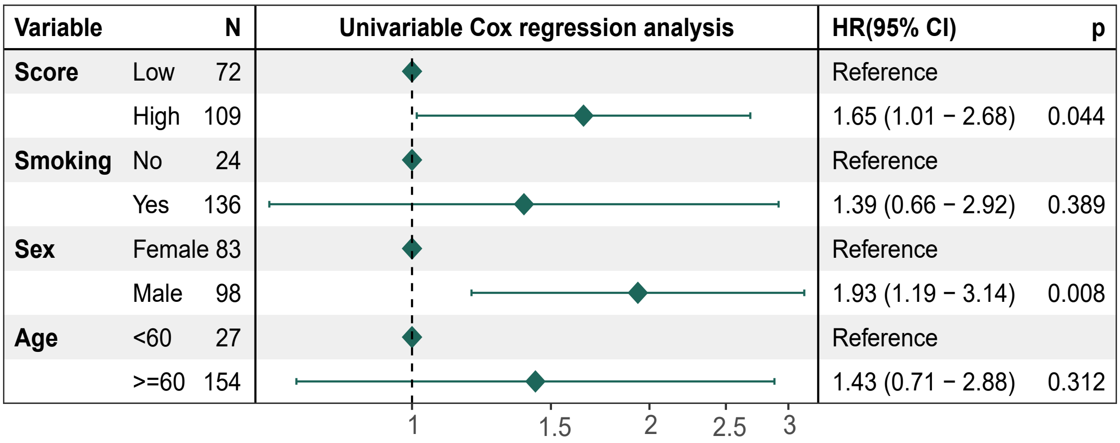
Variable N
Univariable Cox regression analysis
HR(95% CI) p
Score Low 72 Reference
High 109 1.65 (1.01 − 2.68) 0.044
Smoking No 24 Reference
Yes 136 1.39 (0.66 − 2.92) 0.389
Sex Female 83 Reference
Male 98 1.93 (1.19 − 3.14) 0.008
Age <60 27 Reference
>=60 154 1.43 (0.71 − 2.88) 0.312
1
1.5
2
2.5
3

Supplementary Table 3

label	variable	value
Smoking	No	90 (9.08%)
	unknow	35 (3.53%)
	Yes	866 (87.39%)
EGFR	Mut	100 (10.09%)
	unknow	429 (43.29%)
	WT	462 (46.62%)
ALK_eml4	NO	477 (48.13%)
	unknow	471 (47.53%)
	YES	43 (4.34%)
KRAS	Mut	73 (7.37%)
	unknow	372 (37.54%)
	WT	546 (55.10%)
Histological.Type	LUAD	500 (50.45%)
	LUSC	491 (49.55%)
Stage	I/II	785 (79.21%)
	III/IV	194 (19.58%)
	unknow	12 (1.21%)
Age	Age<60	241 (24.32%)
	Age>=60	750 (75.68%)
Sex	Female	397 (40.06%)
	Male	594 (59.94%)

Supplementary Table 4

Characteristics		N=13
Age (Mean±SD)		54.00±10.68
Tumor Size (Mean±SD)		3.75 ± 1.27
Sex (n, %)		
	Male	6(46.15)
	Female	7(53.85)
Histological Typing (n, %)		
	Squamous	10(76.92)
	Adenocarcinoma	3(23.08)
Smoking (n, %)		
	Smoker	9(69.23)
	Never-smoker	4(30.77)
TMN Stage (n, %)		
	I/Ⅱ	8(61.54)
	III/Ⅳ	5(38.46)
Lympho Node Metastasis (n, %)		
	Yes	3(23.08)
	No	10 (76.92)
Distant Metastasis (n, %)		
	Yes	2 (15.38)
	No	11(84.62)
